# Anakinra for palmoplantar pustulosis: results from a randomized, double‐blind, multicentre, two‐staged, adaptive placebo‐controlled trial (APRICOT)*

**DOI:** 10.1111/bjd.20653

**Published:** 2021-10-12

**Authors:** S. Cro, V.R. Cornelius, A.E. Pink, R. Wilson, A. Pushpa‐Rajah, P. Patel, A. Abdul‐Wahab, S. August, J. Azad, G. Becher, A. Chapman, G. Dunnill, A.D. Ferguson, A. Fogo, S.A. Ghaffar, J.R. Ingram, S. Kavakleiva, E. Ladoyanni, J.A. Leman, A.E. Macbeth, A. Makrygeorgou, R. Parslew, A.J. Ryan, A. Sharma, A.R. Shipman, C. Sinclair, R. Wachsmuth, R.T. Woolf, A. Wright, H. McAteer, J.N.W.N. Barker, A.D. Burden, C.E.M. Griffiths, N.J. Reynolds, R.B. Warren, H.J. Lachmann, F. Capon, C.H. Smith, the APRICOT Study Group, Davide Altamura, Vincent Piguet, Roberto Verdolini, Marc Wallace, Kavitha Sundararaj, Nisha Arujuna, Charlotte Fleming, Ruth Lamb, Jacqueline Dodds, Sonia Baryschpolec, Hywel Cooper

**Affiliations:** ^1^ Imperial Clinical Trials Unit Imperial College London London W12 7RH UK; ^2^ St John’s Institute of Dermatology Guy’s Hospital Guy’s and St Thomas’ NHS Foundation Trust London SE1 9RT UK; ^3^ St George’s University Hospitals NHS Foundation Trust London SW17 0QT UK; ^4^ Poole Hospital NHS Foundation Trust University Hospitals Dorset Poole BH15 2JB UK; ^5^ South Tees Hospitals NHS Foundation Trust Middlesbrough TS4 3BW UK; ^6^ West Glasgow Ambulatory Care Hospital Glasgow G3 8SJ UK; ^7^ Homerton University Hospital London E9 6SR UK; ^8^ Bristol Royal Infirmary Bristol BS2 8HW UK; ^9^ University Hospitals of Derby and Burton NHS Foundation Trust Derby DE22 3NE UK; ^10^ Kingston Hospital Kingston upon Thames KT2 7QB UK; ^11^ Ninewells Hospital and Medical School Dundee DD1 9SY UK; ^12^ Division of Infection and Immunity School of Medicine Cardiff University University Hospital of Wales Cardiff CF14 4XN UK; ^13^ Royal Lancaster Infirmary Lancaster LA1 4RP UK; ^14^ Russells Hall Hospital Dudley DY1 2HQ UK; ^15^ Kings Park Hospital Stirling FK7 9JH UK; ^16^ Norfolk and Norwich University Hospitals NHS Foundation Trust Norwich NR4 7UY UK; ^17^ Liverpool University Hospitals NHS Foundation Trust Liverpool L9 7AL UK; ^18^ King’s College Hospital London SE5 9RS UK; ^19^ Nottingham University Hospitals NHS Trust Nottingham NG7 2UH UK; ^20^ Portsmouth Hospitals Universities NHS Trust St Mary’s Community Health Campus Portsmouth PO3 6AD UK; ^21^ Broomfield Hospital Chelmsford CM1 7ET UK; ^22^ Royal Devon and Exeter NHS Foundation Trust Exeter EX2 5DW UK; ^23^ Bradford Teaching Hospitals NHS Foundation Trust Bradford BD9 6RJ UK; ^24^ The Psoriasis Association Northampton NN4 7BF UK; ^25^ St John’s Institute of Dermatology School of Basic and Medical Biosciences Faculty of Life Sciences and Medicine King’s College London London SE1 9RT UK; ^26^ Institute of Infection, Immunity and Inflammation University of Glasgow Glasgow G12 8TA UK; ^27^ Dermatology Centre Salford Royal NHS Foundation Trust University of Manchester NIHR Manchester Biomedical Research Centre Manchester M6 8HD UK; ^28^ Institute of Translational and Clinical Medicine Medical School University of Newcastle Department of Dermatology Royal Victoria Infirmary and NIHR Newcastle Biomedical Research Centre Newcastle Hospitals NHS Foundation Trust Newcastle upon Tyne NE2 4HH UK; ^29^ National Amyloidosis Centre University College London London NW3 2PF UK; ^30^ Department of Medical and Molecular Genetics King’s College London London SE1 9RT UK

## Abstract

**Background:**

Palmoplantar pustulosis (PPP) is a rare, debilitating, chronic inflammatory skin disease that affects the hands and feet. Clinical, immunological and genetic findings suggest a pathogenic role for interleukin (IL)‐1.

**Objectives:**

To determine whether anakinra (an IL‐1 receptor antagonist) delivers therapeutic benefit in PPP.

**Methods:**

This was a randomized (1 : 1), double‐blind, two‐staged, adaptive, UK multicentre, placebo‐controlled trial [ISCRTN13127147 (registered 1 August 2016); EudraCT number: 2015‐003600‐23 (registered 1 April 2016)]. Participants had a diagnosis of PPP (> 6 months) requiring systemic therapy. Treatment was 8 weeks of anakinra or placebo via daily, self‐administered subcutaneous injections. Primary outcome was the Palmoplantar Pustulosis Psoriasis Area and Severity Index (PPPASI) at 8 weeks.

**Results:**

A total of 374 patients were screened; 64 were enrolled (31 in the anakinra arm and 33 in the placebo arm) with a mean (SD) baseline PPPASI of 17·8 (10·5) and a PPP investigator’s global assessment of severe (50%) or moderate (50%). The baseline adjusted mean difference in PPPASI favoured anakinra but did not demonstrate superiority in the intention‐to‐treat analysis [–1·65, 95% confidence interval (CI) –4·77 to 1·47; *P* = 0·30]. Similarly, secondary objective measures, including fresh pustule count (2·94, 95% CI –26·44 to 32·33; favouring anakinra), total pustule count (–30·08, 95% CI –83·20 to 23·05; favouring placebo) and patient‐reported outcomes, did not show superiority of anakinra. When modelling the impact of adherence, the PPPASI complier average causal effect for an individual who received ≥ 90% of the total treatment (48% in the anakinra group) was –3·80 (95% CI –10·76 to 3·16; *P* = 0·285). No serious adverse events occurred.

**Conclusions:**

No evidence for the superiority of anakinra was found. IL‐1 blockade is not a useful intervention for the treatment of PPP.

## Abstract 2

**What is already known about this topic?**

- Treatment options for palmoplantar pustulosis include superpotent corticosteroids, phototherapy, acitretin, methotrexate and ciclosporin. However, these have poor evidence for benefit and there is a risk of toxicity with long‐term use.
- Anakinra is a recombinant interleukin (IL)‐1 receptor antagonist that blocks the activity of IL‐1α and IL‐1β, two cytokines repeatedly linked to neutrophil activation and extravasation.
- A therapeutic benefit of anakinra has been shown in neutrophilic dermatoses and conditions that manifest with skin pustulation.

**What does this study add?**

- Anakinra was not significantly superior to placebo at 8 weeks in objective investigator‐assessed and patient‐reported measures.
- A greater proportion of participants in the anakinra group strongly agreed that the treatment was worthwhile.
- The safety profile of anakinra was consistent with previous studies.
- This is one of the largest randomized controlled trials for this rare condition, providing important data on its natural history and change in disease severity over time.

**Linked Comment:** S.N. Lo and J.F. Thompson. Br J Dermatol 2022; 186:205–206.

Plain language summary available online

## Graphical abstract

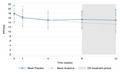

Palmoplantar pustulosis (PPP) is a rare, chronic, inflammatory skin disease characterized by sterile neutrophilic pustules on the palms and soles.^1^, ^2^ It is associated with plaque psoriasis in about 20% of cases.^3^ Often accompanied by fissures, pruritus and a burning sensation, the disease is painful and disabling, and can severely impact on patients’ quality of life.^4^, ^5^, ^6^ Management options are profoundly limited. Commonly used treatments include superpotent corticosteroids, phototherapy, acitretin, methotrexate and ciclosporin, for which there is poor evidence of benefit and the risk of significant toxicity with long‐term use.^7^ Equally, the biological therapies, particularly those targeting the canonical interleukin (IL)‐23/IL‐17 pathway, that deliver such impressive clearance rates in plaque psoriasis only show modest benefit in PPP, with two randomized controlled trials (RCTs) reporting data for secukinumab and guselkumab, respectively.^8^, ^9^

Anakinra is a recombinant IL‐1 receptor antagonist (IL‐1Ra) that is currently licensed for the treatment of rheumatoid arthritis and cryopyrin‐associated periodic syndromes. It blocks the activity of IL‐1α and IL‐1β, two cytokines that have been repeatedly linked to neutrophil activation and extravasation. In keeping with these observations, anakinra has shown therapeutic benefit in neutrophilic dermatoses and in conditions characterized by skin pustulation.^10^ The latter include deficiency of IL‐1Ra, generalized pustular psoriasis caused by *IL36RN* mutations, acrodermatitis continua of Hallopeau and amicrobial pustulosis of the folds.^11^, ^12^, ^13^, ^14^, ^15^ Anakinra also showed efficacy in patients who presented with PPP in the context of SAPHO syndrome (synovitis, acne, pustulosis, hyperostosis, osteitis).^16^

We therefore designed this randomized, double‐blind, multicentre, two‐staged, adaptive, placebo‐controlled trial to determine the efficacy of anakinra for the treatment of adults with PPP.

## Patients and methods

### Study design and participants

Enrolment into APRICOT (Anakinra for Pustular Psoriasis: Response in a Controlled Trial) was conducted across 16 sites in England, Scotland and Wales between October 2016 and January 2020. Participants were randomly allocated to 8 weeks of treatment with anakinra or placebo. Study visits for outcome assessments occurred at weeks 1, 4, 8 and 12. The trial included two stages and an adaptive element. Stage one (the first 24 participants) compared treatment groups to ensure proof of concept and select the primary outcome for stage two [see Appendix S3 (Supporting Information) for the full details of stage 1]. Full details on the trials methods have been published previously.^17^ Ethical approval was granted by London Dulwich Research Ethics Committee (16/LO/0436).

In brief, eligible participants were aged ≥ 18 years with a diagnosis of PPP with disease of a sufficient severity to require systemic therapy, duration > 6 months not responding to topical therapy (including potent corticosteroids), active pustules on the palms and/or soles, at least a moderate score on the Palmoplantar Pustulosis Investigator’s Global Assessment (PPP‐IGA), women of childbearing potential on adequate contraception and not pregnant or breastfeeding, and able to give written informed consent to participate. The list of exclusions can be found in the trial protocol and included the use of therapies with potential or known efficacy in PPP during or within stipulated time frames before treatment initiation (see Table S1 in Appendix S3).^17^ After the trial started, two exclusions were added as a precaution, following new information in the Summary of Product Characteristics^18^: (i) thrombocytopenia and (ii) diagnosis (or historic diagnosis) of childhood or adult‐onset Still disease. Part way through the trial an open‐label extension was added and offered to all who had completed the treatment period, primarily to enhance recruitment, and will be reported elsewhere (Cro *et al*., submitted).

### Patient involvement

A patient and public‐involvement group including people with pustular psoriasis and representation from the UK’s main organization for patients with psoriasis (Psoriasis Association) provided input and support into study design (prioritizing the study question, use of placebo and 8‐week treatment duration), delivery (patient information and recruitment communications), interpretation of the results and communication of outcomes.

### Randomization and blinding

To ensure allocation concealment, participants were randomised (1 : 1), using a secure web‐based randomisation system hosted by King’s College London Clinical Trials Unit, to anakinra or placebo. The allocation sequence was generated using blocked randomization stratified by centre. Throughout the trial, participants, research nurses, treating physicians and independent outcome assessors were blind to treatment assignment. To avoid inadvertent unblinding (injection site reactions are common and can be severe with anakinra), independent assessors performed the outcome assessment in silence, and with only the trial participant’s hands and feet exposed.

### Interventions

Participants allocated to the active group received anakinra (Kineret; SOBI, Stockholm, Sweden) 100 mg/0·67 mL daily through self‐administered subcutaneous (SC) injection. The placebo group received identical matched syringes containing 0·67 mL vehicle solution only. Participants self‐administered a daily SC injection of the product for 8 weeks.

Adherence was measured using a daily text message reminder that required participants to confirm the treatment had been taken. Participants were also instructed to complete an injection diary card and asked at each visit for a record of their daily usage.

Emollient therapy was permitted throughout the trial. Potent corticosteroid dispensed as ‘rescue’ therapy was recorded by the study team. Prohibited therapies included ultrapotent topical corticosteroids, phototherapy and systematic therapies (see Table S2 in Appendix S3). Mild‐to‐moderate corticosteroids were permitted for plaque psoriasis at sites other than the hands and feet. Mild topical corticosteroids and/or antihistamines could be used to treat injection site reactions.

### Outcomes

The primary outcome was the week‐8 Palmoplantar Pustulosis Psoriasis Area and Severity Index (PPPASI),^19^ adjusted for baseline PPPASI (i.e. change PPPASI at week 8). Investigator‐assessed secondary outcomes at 8 weeks included baseline‐adjusted fresh pustule count on the palms and soles, total pustule count on the palms and soles, PPP‐IGA, clear on PPP‐IGA and disease flare (> 50% deterioration in PPPASI). Time to response of PPP (≥ 75% reduction in fresh pustule count) and time to relapse (return to baseline of fresh pustule count) were assessed over 12 weeks. Participant‐assessed secondary outcomes at 8 weeks adjusted for baseline included the Dermatology Life Quality Index (DLQI), Palmoplantar Quality of Life instrument score (PPQoL), patient global assessment (PGA), treatment acceptability evaluated using a 5‐point response scale as to whether the treatment was worthwhile (strongly disagree/disagree/neither agree nor disagree/agree/strongly agree) at week 12 and adherence. Safety outcomes included serious infection, neutropenia, clinically significant changes in other haematological parameters, and renal or liver function. The incidence of adverse events (AEs) was recorded and coded according to MedDRA. Outcomes assessed post‐hoc were ≥ 50% improvement in PPPASI (PPPASI‐50), ≥ 75% improvement in PPPASI (PPPASI‐75) and the PPPASI pustule subscale at 8 weeks.

### Statistical analysis

Sample size was calculated by reference to a standardized effect size, as determined prior to the end of stage 1 when the primary outcome was unknown. A large effect size of 0·9 SDs was selected to be the minimally important difference to detect, as described in the previously published protocol.^17^ To detect 0·9 SD with 90% power, 5% significance level and allowing for 15% withdrawal, a sample size of 64 (32 per arm) was required.

Analysis was conducted blinded to subgroup (i.e. group A vs. group B), in accordance with APRICOT’s statistical analysis protocol.^20^ The main analysis was based on the intention‐to‐treat (ITT) principle, to estimate the effect of the 8‐week treatment policy (see Appendix S3 for a description of estimands).^21^ For the primary outcome, a linear mixed‐effect model estimated the mean between‐group difference in PPPASI at 8 weeks. Missing responses were assumed to be missing at random. Sensitivity analysis explored missing‐not‐at‐random assumptions.^22^ Supplementary analysis, using the methods described in Appendix S3, explored the treatment effect (i) if rescue therapy was not available; (ii) if rescue and prohibited therapy was not available; (iii) if all topical therapy was not available; and (iv) the complier average casual effect (CACE) were calculated. The CACE analysis retains the initial randomization and provides an estimate of the treatment effect for individuals who would be able to comply with ≥ 50–90% of the prescribed daily injections by comparing the compliers in the anakinra group with the comparable group of compliers in the placebo group. Estimates are presented with 95% confidence intervals (CIs) and *P*‐values. A *P*‐value < 0·05 was interpreted as being statistically significant for the primary outcome. Additional statistical methods are described in Appendix S3.

## Results

### Participant flow

**Figure 1:**
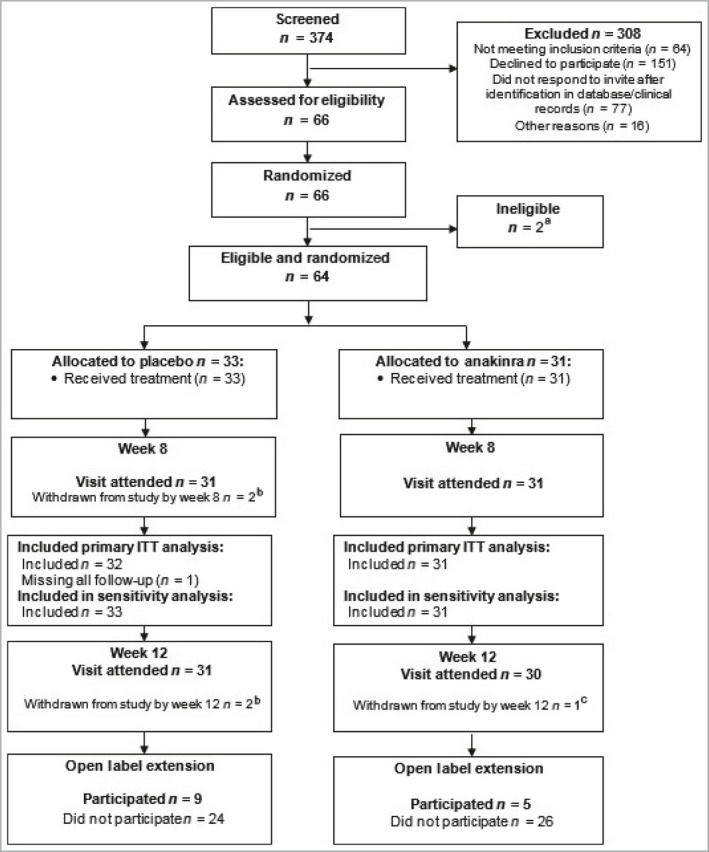
CONSORT flow chart. ITT, intention to treat. ^a^Two participants were randomized in error and were subsequently found to be ineligible. They were not offered treatment and were immediately withdrawn from the study, and excluded from all analyses. ^b^One participant withdrew from the trial in week 1. One participant was lost to follow‐up and withdrawn after week 4. ^c^One participant withdrew at week 8. Numbers withdrawn from the trial are cumulative.

From October 2016 to January 2020, 374 patients were screened and 64 eligible participants were enrolled; 33 were randomized to placebo and 31 to anakinra (Figure 1). Trial participants had a mean (SD) age of 50·8 (12·7) years, and were predominantly white females, and current or ex‐smokers. Baseline characteristics, including disease characteristics, were well balanced across treatment groups, with a mean (SD) baseline PPPASI of 17·8 (10·5) (Table 1).

**Table 1:** Baseline characteristics of participants in APRICOT by treatment group

Baseline demographic	Placebo (*n* = 33)	Anakinra (*n* = 31)	Total (*n* = 64)
Mean (SD) age (years)	51·7 (13·6)	49·9 (11·9)	50·8 (12·7)
Sex			
Male	6 (18)	4 (13)	10 (16)
Female	27 (82)	27 (87)	54 (84)
Ethnicity			
White	31 (94)	28 (90)	59 (92)
Asian/Asian British	1 (3)	1 (3)	2 (3)
Black/Black British	0 (0)	1 (3)	1 (2)
Chinese/Japanese/Korean/Indochinese	0 (0)	1 (3)	1 (2)
Other	1 (3)	0 (0)	1 (2)
Smoker			
Current smoker	19 (58)	16 (52)	35 (55)
Ex‐smoker	9 (27)	12 (39)	21 (33)
Nonsmoker	5 (15)	3 (10)	8 (13)
PPPASI			
Mean (SD)	18·0 (10·4)^a^	17·5 (10·8)	17·8 (10·5)
Median (IQR)	15·9 (10·4–21·3)	15·4 (11·7–20·7)	15·6 (10·6–21·0)
Fresh pustule count (palms and soles)			
Mean (SD)	36·1 (33·1)	39·8 (46·3)^a^	37·9 (39·6)
Median (IQR)	28·0 (18·0–45·0)	25·5 (11·0–58·0)	27·0 (15·0–49·0)
Fresh pustule count (soles)			
Mean (SD)	25·9 (23·4)	29·6 (43·2)^a^	27·7 (34·1)
Median (IQR)	23·0 (4·0–36·0)	15·0 (5·0–37·0)	19·0 (4·0–37·0)
Fresh pustule count (palms)			
Mean (SD)	10·2 (19·2)	10·2 (16·5)^a^	10·2 (17·8)
Median (IQR)	2·0 (0·0–13·0)	2·5 (0·0–13·0)	2·0 (0·0–13·0)
Total pustule count (palms and soles)			
Mean (SD)	116·9 (96·4)	154·3 (198·7)^a^	134·7 (153·7)
Median (IQR)	97·0 (45·0–169·0)	89·0 (45·0–157·0)	95·0 (45·0–169·0)
PPP‐IGA^b^			
Moderate	16 (48)	16 (52)	32 (50)
Severe	17 (52)	15 (48)	32 (50)
Participant global assessment			
Almost clear	0 (0)	2 (6)	2 (3)
Mild	3 (9)	3 (10)	6 (9)
Moderate	14 (42)	14 (45)	28 (44)
Severe	13 (39)	7 (23)	20 (31)
Very severe	3 (9)	5 (16)	8 (13)
Mean (SD) DLQI	13·9 (7·2)	15·1 (7·0)	14·5 (7·1)
PASI^c^			
Mean (SD)	2·1 (5·4)	1·1 (1·6)	1·6 (4·1)
Median (IQR)	0·0 (0·0–1·8)	0·2 (0·0–1·6)	0·0 (0·0–1·6)
Mean (SD) PPQoL	46·4 (13·8)	45·5 (14·8)	46·0 (14·2)
EQ‐5D utility score			
Mean (SD)	0·37 (0·43)	0·47 (0·35)	0·42 (0·40)
Median (IQR)	0·62 (0·09–0·73)	0·62 (0·16–0·73)	0·62 (0·09–0·73)
EQ‐5D VAS			
Mean (SD)	57·7 (27·7)	68·4 (18·3)^d^	62·5 (24·4)
Median (IQR)	65·0 (45·0–80·0)	75·0 (55·0–80·0)	70·0 (50·0–80·0)

Data are *n* (%) unless otherwise stated. DLQI, Dermatology Life Quality Index; EQ‐5D, EuroQol five‐dimension health‐related quality of life instrument; IQR, interquartile range; PASI, Psoriasis Area and Severity Index; PPPASI, Palmoplantar pustulosis Psoriasis Area and Severity Index; PPP‐IGA, Palmoplantar Pustulosis Investigator Global Assessment; PPQoL, Palmoplantar Quality of Life instrument score; VAS, visual analogue scale. ^a^One participant was missing this outcome in the indicated treatment group. ^b^Worse PPP‐IGA rating from two independent assessors. ^c^PASI measurements were available for 19 participants in the placebo group and 16 in the anakinra group. ^d^Four participants in the anakinra group were missing baseline EQ‐5D VAS.

### Withdrawals, adherence and use of nontrial treatment

Over the 8‐week treatment period, six (18%) placebo and five (16%) anakinra participants permanently withdrew from treatment. Retention in the study was high: 97% at week 8 and 95% at week 12 (Figure 1). However, overall, adherence to treatment fell over time in both arms from a mean (SD) number of injections over week 1 of 6·1 (1·9) for placebo and 6·7 (0·6) for anakinra to 4·8 (3·1) and 5·3 (2·7), respectively, over week 8; 81% of the anakinra group took ≥ 50% of daily injections but only 48% took > 90% of daily injections (see Tables S6 and S7 in Appendix S3).

There was no clinically significant difference between treatment arms with respect to the use of rescue therapy or prohibited therapy (three in each group; see Tables S8–S11 in Appendix S3). Other topical treatments used at sites other than areas affected by PPP were used more in the anakinra group [*n* = 13 (42%)] than in the placebo group [*n* = 7 (21%)] reflecting use for anakinra‐related injection site reactions (see Tables S12 and S13 in Appendix S3).

### Primary outcome

**Figure 2:**
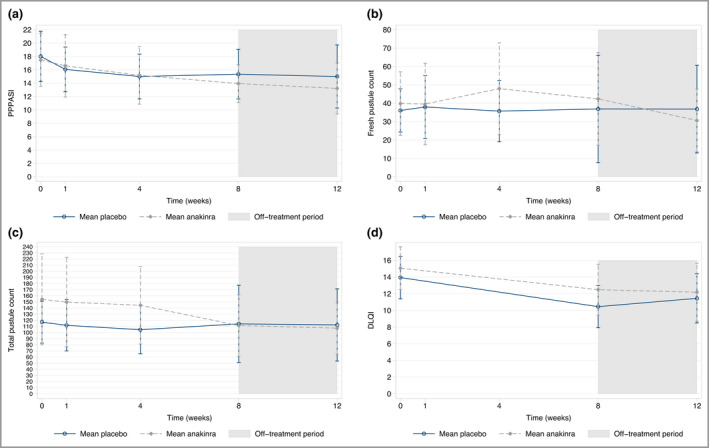
(a) Palmoplantar Pustulosis Psoriasis Area and Severity Index (PPPASI); (b) fresh pustule count; (c) total pustule count; and (d) Dermatology Life Quality Index (DLQI) over the 12‐week follow‐up period. Error bars represent 95% confidence intervals.

In the ITT analysis the mean difference in PPPASI at week 8 was in favour of anakinra but did not demonstrate superiority (–1·65, 95% CI –4·77 to 1·47; *P* = 0·30) (Figure 2, Table 2). Sensitivity analyses under alternative missing data assumptions supported the primary result (see Table S14 in Appendix S3). The mean difference in PPPASI at week 12 for anakinra vs. placebo was –2·42 (95% CI –5·97 to 1·13; *P* = 0·182).

**Table 2:** Primary and secondary APRICOT outcomes

Outcome	Placebo		Anakinra		Unadjusted MD: anakinra–placebo (95% CI)	Adjusted mean difference: anakinra–placebo (95% CI)	*P*‐value
	Mean (SD)	*n*	Mean (SD)	*n*			
Primary outcome							
PPPASI (week 8)^a^	15·4 (10·1)	31	13·9 (7·4)	29	–1·4 (–6·0 to 3·2)	–1·65 (–4·77 to 1·47)	0·300
Secondary outcomes							
Fresh pustule count (week 8) palm + sole	36·9 (79·5)	31	42·4 (65·1)	28	5·5 (–32·6 to 43·6)	2·94 (–26·44 to 32·33)	0·844
Fresh pustule count (week 8) palm	7·0 (14·7)	31	10·8 (19·2)	29	3·9 (–4·9 to 12·7)	4·07 (–5·78 to 13·92)	0·418
Fresh pustule count (week 8) sole	29·9 (69·1)	31	31·4 (61·2)	28	1·5 (–32·7 to 35·7)	–1·42 (–27·33 to 24·48)	0·914
Total pustule count (week 8)	114·2 (171·8)	31	111·4 (129·3)	28	–2·8 (–82·7 to 77·2)	–30·08 (–83·20 to 23·05)	0·267
PASI	0·8 (1·7)	16	0·9 (1·1)	15	0·0 (–1·0 to 1·1)	–0·41 (–0·96 to 0·15)	0·151
PPQoL	40·2 (16·0)	31	41·4 (13·9)	31	1·2 (–6·4 to 8·8)	1·27 (–3·04 to 5·57)	0·564
DLQI	10·5 (6·9)	31	12·5 (8·3)	31	2·0 (–1·9 to 5·9)	0·52 (–2·04 to 3·07)	0·692
EQ‐5D‐3L	0·6 (0·4)	31	0·5 (0·4)	31	0·0 (–0·2 to 0·2)	–0·09 (–0·23 to 0·06)	0·227
	***n*** **(%)**	***n***	***n* (%)**	***n***	**Unadjusted difference in proportion (%): anakinra–placebo (95% CI)**	**Adjusted OR (95% CI)**	***P*‐value**
PPPASI‐50 (week 8)^b^	5 (16)	31	6 (21)	29	4·6 (–15·1 to 24·2)	1·68 (0·35–8·19)	0·520
PPPASI‐75 (week 8)^b^	1 (3)	31	0 (0)	29	–3·2 (–9·4 to 3·0)	Unestimable	
PPPASI pustule subscale palm (week 8)		31		29			
None	14 (45)		11 (37)			2·51 (0·56–11·28)	0·231
Slight	10 (32)		9 (30)				
Moderate	5 (16)		8 (27)				
Severe	2 (6)		2 (7)				
Very severe	0 (0)		0 (0)				
PPPASI pustule subscale soles (week 8)		31		29			
None	3 (10)		2 (7)			1·63 (0·49–5·46)	0·426
Slight	6 (19)		8 (28)				
Moderate	11 (35)		8 (28)				
Severe	9 (29)		9 (31)				
Very severe	2 (6)		2 (7)				
PPP‐IGA (week 8)		28		30		0·54 (0·13–2·19)	0·384
Almost clear	2 (7)		1 (3)				
Mild	4 (14)		6 (20)				
Moderate	12 (43)		17 (57)				
Severe	10 (36)		6 (20)				
Disease flare (>50% deterioration in PPPASI)	4 (13)	31	2 (7)	29	–6·0 (–20·98 to 8·97)	0·55 (0·08–3·71)	0·542
PGA (week 8)		30		31		1·39 (0·41–4·70)	0·597
Clear	1 (3)		0 (0)				
Nearly clear	3 (10)		3 (10)				
Mild	4 (13)		5 (16)				
Moderate	11 (37)		11 (35)				
Severe	10 (33)		10 (32)				
Very severe	1 (3)		2 (6)				
						**Adjusted HR (95% CI)**	***P*‐value**
Time to response (75% reduction fresh pustule count)	15 (48)	31	13 (43)	30		0·58 (0·22–1·50)	0·263
Time to relapse (return to baseline fresh pustule count)	19 (61)	31	20 (67)	30		0·94 (0·50–1·7)	0·853

CI, confidence interval; DLQI, Dermatology Life Quality Index; EQ‐5D‐5L, EuroQol five‐dimension, five‐level, health‐related quality of life instrument; HR, hazard ratio; MD, mean difference; OR, odds ratio; PASI, Psoriasis Area and Severity Index; PGA, Patient’s Global Assessment; PPPASI, Palmoplantar Pustulosis Psoriasis Area and Severity Index; PPPASI‐50, ≥ 50% reduction in PPPASI; PPPASI‐75, ≥ 75% reduction in PPPASI; PPP‐IGA, Palmoplantar Pustulosis Investigator Global Assessment; PPQoL, Palmoplantar Quality of Life instrument score. ^a^Complier average causal effect for PPPASI: ≥ 50% injections –3·37 (–6·98 to 0·23; *P* = 0·07) and ≥ 90% injections –5·53 (–11·39 to 0·32; *P* = 0·07). ^b^Post‐hoc outcome. In both groups, no participants experienced serious infection of neutropenia.

### Impact of adherence and nontrial treatments on primary outcome

Using CACE analysis, the estimated mean treatment difference for a complier, defined as an individual taking ≥ 50% of daily injections (81% of the anakinra group), was –2·30 (95% CI –6·54 to 1·93; *P* = 0·287). The CACE was similar for ≥ 60% to ≥80% adherence (data not shown). For ≥ 90% adherence (48% in the anakinra group) the CACE was –3·80 (95% CI –10·76 to 3·16; *P* = 0·285).

The treatment effect, in the absence of rescue and prohibited therapy was similar (–2·09, 95% CI –8·47 to 4·29; *P* = 0·518). Additional supplementary analyses similarly demonstrated no benefit (see Tables S15–S17 in Appendix S3).

### Secondary outcomes

Anakinra did not demonstrate superiority vs. placebo in any of the secondary outcomes, including objective disease severity assessments, patient‐assessed disease severity (PGA) or impact (DLQI and PPQoL) (see Table 2 and Figure 2). A total of 12 of 29 participants (41%) strongly agreed that the treatment was worthwhile in the anakinra group vs. four of 28 (14%) in the placebo group (see Table S18).

### Safety

**Figure 3:**
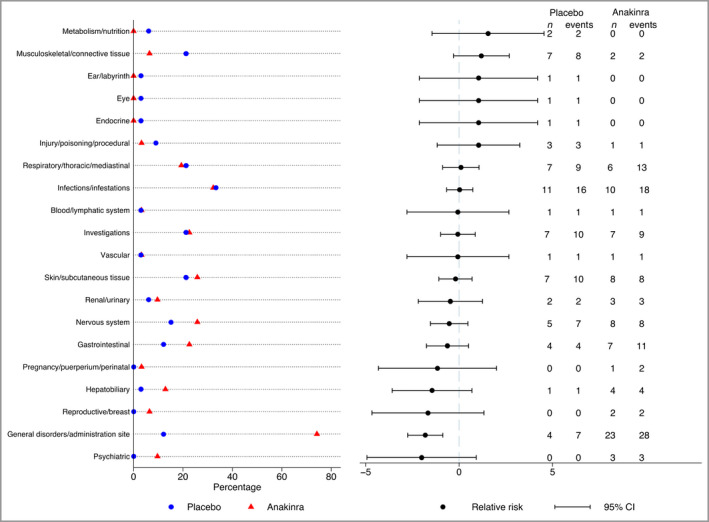
Adverse events by MedDRA (Medical Dictionary for Regulatory Activities) system organ class. CI, confidence interval.

In accordance with the known profile of anakinra, neutrophil counts, total white cell counts (WCC) and platelets were lower in the anakinra group but did not reach clinical significance with mean difference in week 8 change (neutrophil count –0·9, 95% CI –1·7 to 0·0; WCC –1·0, 95% CI –2·0 to 0·0; platelets –25·3, 95% CI –39·6 to –11·1) (see Table S19 in Appendix S3). Across treatment groups, no participants experienced a serious infection, neutropenia or other serious AE. A total of 84 nonserious AEs in 26 participants were reported in the placebo group vs. 114 events in 29 participants in the anakinra group. There was a higher number of injection site reactions in the anakinra group (20 events in 19 participants) than in the placebo group (one event in one participant), explaining the higher number of MedDRA events termed ‘general disorders and administration site conditions’ in the anakinra group (Figure 3). A full listing of AEs is given in Table S20 (see Appendix S3).

## Discussion

This novel, two‐stage adaptive trial aimed to address the hypothesis that IL‐1 blockade benefits PPP. We compared the IL‐1Ra anakinra to placebo in a double‐blind RCT and comprehensively evaluated efficacy and safety after 8 weeks of treatment using objective investigator‐assessed and patient‐reported measures. We found no evidence for the superiority of anakinra. There were more injection site reactions in the anakinra group, but otherwise the frequency of AEs was comparable to placebo.

Some of the findings in this trial raise the possibility that anakinra could have a treatment effect in PPP. Firstly, a greater proportion of participants in the anakinra group strongly agreed that the treatment was worthwhile (41%) vs. the placebo group (14%). This perceived benefit could be due to an effect on disease severity or an impact that we did not identify, despite comprehensively assessing objective and patient‐reported measures. Alternatively, it could be that anakinra exerts a systemic anti‐inflammatory effect that improved well‐being or reduced neuroinflammation, and positively impacted on fatigue (although there was no difference in C‐reactive protein levels between the two arms).^23^ Secondly, the CACE analysis estimate suggested that poor adherence may have contributed to the lack of observed benefit. This is perhaps not unexpected given the daily injection schedule. Among all randomised participants the PPPASI treatment effect was –1·65, whereas those who had at least 90% of prescribed treatment (approximately half) had just over double the effect size (–3·80); this corresponds to a 21% reduction in baseline PPPASI and is just outside the calculated minimally important clinical difference in PPPASI (estimated between 4 and 5·25; see Appendix S3). Thirdly, although not significant, the treatment effect in PPPASI was maintained and marginally increased at 12 weeks (4 weeks post‐treatment cessation). Recent trials with other interventions in PPP are consistent with the notion that longer treatment duration may be necessary to deliver clinical benefit.^24^, ^25^ A phase II RCT of guselkumab that showed no significant change in PPPASI after 8 weeks reported benefit at week 16 that improved consistently through to week 52,^8^ and a phase IIIb RCT of secukinumab showed no difference in the primary PPPASI‐75 outcome at 16 weeks but a trend towards benefit up to week 52.^9^

Based on these observations, and the shape of treatment response graph, it is thus conceivable that a larger trial of longer duration, higher anakinra dose and/or improved adherence may have identified a significant effect of anakinra. The treatment duration in our trial was limited to 8 weeks to balance (uncertain) patient benefit and the importance of the research question, against known harms (patients receiving placebo have no opportunity for clinical benefit and all patients run the risk of poorly controlled disease for the duration of the study, plus the burden of self‐administered, daily SC injections commonly associated with injection site reactions, study visits and blood investigations). Early proof‐of‐concept data in generalized (*n* = 4) and localized forms of pustular psoriasis (acrodermatitis of Halopeau, as well as PPP; *n* = 3) available at the time of the study design indicated a rapid resolution of pustules (i.e. within days).^12^, ^13^, ^14^, ^26^, ^27^ We therefore hypothesized that we would see an effect on the pustular element of the disease by 8 weeks. We also sought input from our PPI group, and the collective opinion was that 8 weeks was the maximum reasonable duration of treatment given the daily injections and study design. To minimize safety concerns, we used the dose of anakinra approved for use in licensed indications. Adherence was perhaps lower than expected given our proactive text reminder strategy but is likely to be even lower in clinical practice. Thus, overall, in the context of our robust primary endpoint and lack of observed benefit detected with any of the secondary outcomes, if anakinra exerts some effect in PPP, we are confident that it is unlikely to be clinically relevant. We have answered the question for an 8‐week treatment policy, but whether there is a benefit for those who adhere to the treatment for a longer duration remains unanswered.

Given the absence of benefit with anakinra, these findings also suggest that the pustular phenotype observed in PPP may not be driven by the same IL‐1 family cytokines (IL‐1α/β, IL‐36α/β/γ) that are abnormally active in clinically related conditions. In fact, we have shown that the demographic and genetic features of PPP are entirely distinct from those underlying generalized pustular psoriasis.^28^ Likewise, Liang *et al*. have reported a limited overlap between the genes that are overexpressed in the acral and generalized forms of pustular psoriasis.^29^ Finally, clinical trials have shown that IL‐36 blockade ameliorates the symptoms of generalized pustular psoriasis but shows limited efficacy in PPP.^30^, ^31^, ^32^ In this context, further studies of the genetic and immunological basis of PPP may be required to identify disease‐specific therapeutic targets.

The PPP clinical phenotype does vary between individuals in terms of sites involved, extent, size and number of pustules – variation that is reflected, to some degree, in the wide range of fresh pustules and PPPASI subscores reported in our trial, and as also discussed during the development of the European consensus statement on pustular phenotypes.^1^ Better understanding of the molecular subtypes and roles of environmental triggers that presumably contribute to this variation may offer an opportunity for more targeted, and therefore effective, interventions.

This is one of the largest RCTs in PPP, providing robust evidence, and our follow‐up rates were high. We have established a large study population recallable for future trials, and provide important data on the natural history of PPP and change in disease severity over time using various disease severity scores.

To facilitate retention and reflect clinical practice, rescue therapy with potent corticosteroids was allowed. However, this had a minimal impact on the trial results, only increasing the size of the treatment effect in favour of anakinra by a small amount.

Improvements in outcomes were seen in both treatment groups over time, consistent with trends seen in other recent placebo‐controlled trials of biologics in PPP.^8^, ^9^ It cannot be ruled out that there was some selection towards less severe or unstable patients entering the trial given the study was placebo controlled and had a required washout period. Other limitations included the sample size, which was calculated to detect a large effect size due to being calculated prior to the confirmation of the primary outcome for stage 2. The small sample size meant that estimates for some of the uncommon secondary outcomes lacked precision. We selected anakinra as our preferred IL‐1 blocker because, uniquely, it blocks both IL‐1α and IL‐1β, it has a rapid onset of action and established safety profile (> 70 000 patient‐years exposure), there was early evidence of benefit in pustular psoriasis and has the lowest drug acquisition costs. However, the requirement for daily injections along with the injection site reactions may have negatively influenced compliance. The use of IL‐1 blockers such as rilanocept or canakinumab, which require less frequent administration (weekly and 8 weekly, respectively) may have been associated with better compliance.

An 8‐week anakinra treatment policy was not superior to placebo, meaning that IL‐1 blockade, using anakinra, is unlikely to deliver important clinical utility. These findings also suggest that the IL‐1 family cytokines are not the major disease mediators in PPP. This condition remains an area of high unmet need and further research is required to identify new drug targets.

## Author Contribution

**Suzie Cro:** Formal analysis (lead); Methodology (supporting); Writing‐original draft (lead). **V Cornelius:** Conceptualization (supporting); Formal analysis (equal); Funding acquisition (equal); Methodology (equal); Writing‐review & editing (equal). **Andrew Pink:** Investigation (equal); Writing‐review & editing (equal). **Rosemary Wilson:** Project administration (equal). **Angela Pushpa‐Rajah:** Project administration (equal). **Prakash Patel:** Project administration (equal); Writing‐review & editing (equal). **Alya Abdul‐Wahab:** Investigation (equal). **Suzannah August:** Investigation (equal). **Jaskiran Azad:** Investigation (equal). **Gabrielle Becher:** Investigation (equal). **Anna Chapman:** Investigation (equal). **Giles Dunnill:** Investigation (equal). **Adam David Ferguson:** Investigation (equal). **abigail fogo:** Investigation (equal). **Sharizan Abdul Ghaffar:** Investigation (equal). **John R Ingram:** Investigation (equal). **Svetlana Kavaklieva:** Investigation (equal). **Evmorfia Ladoyanni:** Investigation (equal). **Joyce A Leman:** Investigation (equal). **Abby Macbeth:** Investigation (equal). **Areti Makrygeorgou:** Investigation (equal). **richard parslew:** Investigation (equal). **Aisling J Ryan:** Investigation (equal). **Ashish Sharma:** Investigation (equal). **Alexa Rose Shipman:** Investigation (equal). **Catriona Sinclair:** Investigation (equal). **Rachel Wachsmuth:** Investigation (equal). **Richard T Woolf:** Investigation (equal). **Andrew Wright:** Investigation (equal). **Helen McAteer:** Funding acquisition (equal); Methodology (equal); Writing‐review & editing (equal). **Jonathan N W N Barker:** Funding acquisition (equal); Methodology (equal); Writing‐review & editing (equal). **David Burden:** Funding acquisition (equal); Methodology (equal); Writing‐review & editing (equal). **Christopher Ernest, Maitland Griffiths:** Funding acquisition (equal); Methodology (equal); Writing‐review & editing (equal). **Nick Reynolds:** Funding acquisition (equal); Investigation (equal); Methodology (equal); Writing‐review & editing (equal). **Richard B Warren:** Funding acquisition (equal); Investigation (equal); Methodology (equal); Writing‐review & editing (equal). **Helen J Lachmann:** Funding acquisition (equal); Methodology (equal); Writing‐review & editing (equal). **Francesca Capon:** Data curation (equal); Formal analysis (equal); Funding acquisition (equal); Investigation (equal); Methodology (equal); Writing‐review & editing (equal). **Catherine H. Smith:** Conceptualization (lead); Funding acquisition (lead); Investigation (lead); Methodology (equal); Writing‐review & editing (lead).

## Supporting information


**Appendix S1** Conflicts of interest.

**Appendix S2** APRICOT list of site principal investigators and PIC site investigators.

**Appendix S3** Additional methods and results (including Tables S1–S22).

**Powerpoint S1** Journal Club Slide Set.

## The APRICOT Study group includes:

Dr Davide Altamura (Broomfield Hospital)

Professor Vincent Piguet (University Hospital of Wales)

Dr Roberto Verdolini (The Princess Alexandra Hospital NHS Trust)

Dr Marc Wallace (Addenbrooke's Hospital)

Dr Kavitha Sundararaj (Queen Elizabeth Hospital)

Dr Nisha Arujuna (Kingston Hospital)

Dr Charlotte Fleming (St George's University Hospitals NHS Foundation Trust)

Dr Ruth Lamb (St George's University Hospitals NHS Foundation Trust)

Jacqueline Dodds (South Tees Hospitals NHS Foundation Trust)

Sonia Baryschpolec (Portsmouth Hospitals NHS Trust)

Dr Hywel Cooper (Portsmouth Hospitals NHS Trust)
