# Supplementary material for: Anakinra for palmoplantar pustulosis: results from a randomized, double‐blind, multicentre, two‐staged, adaptive placebo‐controlled trial (APRICOT)
Source: Br J Dermatol. 2021 Oct 12;186(2):245–56. doi: 10.1111/bjd.20653 (PMC9255857; doi:10.1111/bjd.20653)
Supplement: Supplementary file 1 — Appendix S1 Conflicts of interest. [file BJD-186-245-s002.docx]

**Appendix S1**

**Conflicts of interest**

Dr. Cro reports grants from the National Institute of Health Research during the conduct of the study (NIHR advanced fellowship: NIHR30093).

Dr. Capon reports grants from Boehringer-Ingelheim and consultancy fees from AnaptysBio outside the submitted work.

Professor Barker reports personal fees from Amgen, personal fees from Almirall, grants and personal fees from Abbvie, personal fees from Celgene, personal fees from Novartis, grants and personal fees from Lilly, grants and personal fees from Boehringer Ingelheim, personal fees from Bristol Myers Squibb, grants and personal fees from Janssen, personal fees from Sun Pharma and personal fees from UCB outside the submitted work.

Professor Burden reports personal fees from Boehringer Ingelheim, Novartis, Janssen and from Abbvie outside the submitted work.

Professor Griffiths reports grants and personal fees from Almirall, personal fees from Amgen, grants and personal fees from Celgene, personal fees from BMS, pesonal fees from Boehringer Ingelheim, personal fees from LEO Pharma, grants and personal fees from Eli Lilly, grants and personal fees from Janssen, grants and personal fees from Novartis, grants from Sandoz, personal fees from Abbvie, grants from UCB Pharma during the conduct of the study.

Helen McAteer reports grants from Abbvie, Almiral, Amgen, Celgene, Dermal Laboratories, Eli Lilly, Janssen, LEO Pharma, UCB and from T And R Derma outside the submitted work.

Prakash Patel reports grants from Efficacy and Mechanism Evaluation (EME) Programme (Part of the National Institute for Health Research (NIHR)), during the conduct of the study.

Dr. Pink reports grant from AMGEN and personal fees from Abbvie, Lilly, Sanofi, Leo Pharma, Novartis, Almirall, UCB, La-Roche Posay,Janssen and from BMS outside the submitted work.

Professor Reynolds reported receiving lecture fees from AbbVie (to Newcastle University), payment for medical advisory board meeting and lectures fees from Almirall (to Newcastle University), contributing to a clinical trial from AnaptysBio (to Newcastle upon Tyne Hospital), lecture fees from Celgene (to Newcastle University), lecture fees from Janssen (to Newcastle University), grants and serving as a paid member of a medical advisory board from Novartis (to Newcastle University) and lecture fees from UCB Pharma Ltd (to Newcastle University) outside the submitted work.

Professor Warren reports grants and personal fees from AbbVie, grants and personal fees from Almirall, grants and personal fees from Amgen, grants and personal fees from Celgene, grants and personal fees from Janssen, grants and personal fees from Leo, grants and personal fees from Lilly, grants and personal fees from Medac, grants and personal fees from Novartis, grants and personal fees from Pfizer, grants and personal fees from UCB, personal fees from Arena, personal fees from Avillion, personal fees from Boehringer Ingelheim, personal fees from Bristol Myers Squibb and personal fees from Sanofi outside the submitted work.

Rosemary Wilson reports grants from Efficacy and Mechanism Evaluation (EME) Programme (Part of the National Institute for Health Research (NIHR)), during the conduct of the study.

Angela Pushpa-Raja reports grants from Efficacy and Mechanism Evaluation (EME) Programme (Part of the National Institute for Health Research (NIHR)), during the conduct of the study.

Professor Smith reports non personal pecuniary relationships with AbbVie, GlaxoSmithKline, Janssen, Novartis, Pfizer, Regeneron, Sanquin, Qiagen, MedImmune, Celgene, LEO Pharma, UCB Pharma, Sanofi, Boehringer Ingleheim and grants from Boehringer Ingleheim outside the submitted work.

Dr Becher reports grants from UCB, grants from AbbVie, grants from Novartis, grants from Janssen, grants from Almirall, during the conduct of the study; other from UCB, other from AbbVie, other from Novartis, other from Almirall, outside the submitted work; and Participation on a Data Safety Monitoring Board or Advisory Board for AbbVie and Janssen.

Dr Dunnill reports payment for educational lectures and support for attending AAD 2019 from AbbVie, outside the submitted work; and sponsorship of Advanced medical Dermatology meeting with Janssen and participation on Advisory Board for AbbVie and Eli Lilly.

Dr Ferguson reports payment for expert testimony from Leo Pharma and sponsorship for the European Academy Dermatology and Venerology Annual Conference (Madrid 2020) from Janssen.

Dr Ingram reports consulting fees from UCB Pharma, Boehringer Ingelheim, ChemoCentryx and Novartis. Also participation on a Data Safety Monitoring Board for Novartis and participation on Advisory Boards for Viela Bio and Kymera Therapeutics. Also Editor-in-Chief of British Journal of Dermatology.

Dr Ladoyanni reports non - personal pecuniary relations with AbbVie, Almirall, BMS, Galderma, Jansen, Leo Pharma and Novartis. Also has acted in as a consultant/advisor for Leo Pharma and received support from AbbVie for attending virtual meeting. Also participation on Advisory Board for UCB.

Dr Leman reports contract from LEO Pharma supporting a Scientific Fellow and a consulting fee from Boehringer Ingelheim.

Dr Macbeth reports non-financial, academic collaboration with Pfizer.

Dr Makrygeorgou reports consulting fees from Novartis and payment for educational events from Novartis, UCB, AbbVie, Janssen and Eli Lilly. Also has received support for attending meetings from Novartis, AbbVie and UCB. Also participation on a Data Safety Monitoring Board or Advisory Board for Eli Lilly, Novartis, UCB and AbbVie.

Dr Ryan reports support for attending meetings from Janssen UK and Novartis. Also participation on Advisory Board for AbbVie.

Dr Sinclair reports honoraria for lectures from Leo Pharma and support for attending meetings from AbbVie and Leo Pharma.

Dr Woolf reports payment for educational events from AbbVie, Eli Lilly, Leo Pharma, Janssen-Cilag, Novartis, Sandoz, Sanofi and UCB. Also has received support for attending meetings from AbbVie, Leo Pharma, Sanofi and UCB.

All other authors report no known conflicts of interest.
