# Supplementary material for: Anakinra for palmoplantar pustulosis: results from a randomized, double‐blind, multicentre, two‐staged, adaptive placebo‐controlled trial (APRICOT)
Source: Br J Dermatol. 2021 Oct 12;186(2):245–56. doi: 10.1111/bjd.20653 (PMC9255857; doi:10.1111/bjd.20653)
Supplement: Supplementary file 2 — Appendix S2 APRICOT list of site principal investigators and PIC site investigators. [file BJD-186-245-s003.docx]

# Appendix S2

# APRICOT Study Group

Site Principal Investigators and Participating Sites:

Dr Davide Altamura (Broomfield Hospital)

Dr Suzannah August (Poole Hospital NHS Foundation Trust University Hospitals Dorset)

Dr Gabrielle Becher (West Glasgow Ambulatory Care Hospital)

Dr Giles Dunnill (Bristol Royal Infirmary)

Dr Adam D Ferguson (University Hospitals of Derby and Burton NHS Foundation Trust)

Dr Sharizan Abdul Ghaffar (Ninewells Hospital & Medical School)

Dr John R Ingram (University Hospital of Wales)

Professor Vincent Piguet (University Hospital of Wales)

Dr Svetlana Kavakleiva (Royal Lancaster Infirmary)

Dr Effie Ladoyanni (Russells Hall Hospital)

Dr Joyce A Leman (Queen Margaret Hospital and Victoria Hospital)

Dr Abby E Macbeth (Norfolk and Norwich University Hospitals NHS Foundation Trust) with acknowledgments to Dr Priya Patel, Dr Puran Gurung, David Tomlinson and Joc Keshet-Price (Norfolk and Norwich University Hospitals NHS Foundation Trust)

Dr Areti Makrygeorgou (West Glasgow Ambulatory Care Hospital)

Dr Richard Parslew (Liverpool University Hospitals NHS Foundation Trust)

Dr Andrew Pink (Guy's and St Thomas' NHS Foundation Trust)

Professor Nick Reynolds (Royal Victoria Infirmary, Newcastle upon Tyne NHS Foundation Trust)

Dr Ashish Sharma (Nottingham Circle)

Dr Catriona Sinclair (Broomfield Hospital)

Professor Catherine Smith (Guy's and St Thomas' NHS Foundation Trust)

Dr Roberto Verdolini (The Princess Alexandra Hospital NHS Trust)

Dr Rachel Wachsmuth (Royal Devon and Exeter NHS Foundation Trust)

Dr Marc Wallace (Addenbrooke's Hospital)

Professor Richard Warren (Salford Royal NHS Foundation Trust)

Professor Andrew Wright (Bradford Teaching Hospitals NHS Foundation Trust)

PIC sites and Investigators

Dr Aisling J Ryan (Kings College Hospital)

Dr Anna Chapman and Dr Kavitha Sundararaj (Queen Elizabeth Hospital)

Dr Nisha Arujuna and Dr Abigail Fogo (Kingston Hospital)

Dr Alya Abdul-Wahab, Dr Charlotte Fleming and Dr Ruth Lamb (St George's University Hospitals NHS Foundation Trust)

Dr Jaskiran Azad and Jacqueline Dodds (South Tees Hospitals NHS Foundation Trust)

Sonia Baryschpolec, Dr Hywel Cooper and Dr Alexa R Shipman (Portsmouth Hospitals NHS Trust)
