# Supplementary material for: Anakinra for palmoplantar pustulosis: results from a randomized, double‐blind, multicentre, two‐staged, adaptive placebo‐controlled trial (APRICOT)
Source: Br J Dermatol. 2021 Oct 12;186(2):245–56. doi: 10.1111/bjd.20653 (PMC9255857; doi:10.1111/bjd.20653)
Supplement: Supplementary file 3 — Appendix S3 Additional methods and results (including Tables S1–S22). [file BJD-186-245-s001.docx]

Contents

[1 APRICOT Study Group 1](#_Toc66902113)

[2 Additional methods 2](#_Toc66902114)

[2.1 Washout periods for double-blind RCT 2](#_Toc66902115)

[2.2 Concomitant therapy rules for the double-blind RCT 3](#_Toc66902116)

[2.3 Description of treatment estimands 4](#_Toc66902117)

[2.4 Additional statistical methods for stage 1 6](#_Toc66902118)

[2.5 Additional statistical methods for stage 2 6](#_Toc66902119)

[3 Additional results for stage 1 8](#_Toc66902120)

[4 Additional results for stage 2 11](#_Toc66902121)

[5 Minimum Clinically Important Difference 24](#_Toc66902122)

# APRICOT Study Group

Site Principal Investigators and Participating Sites:

Dr Davide Altamura (Broomfield Hospital)

Dr Suzannah August (Poole Hospital NHS Foundation Trust University Hospitals Dorset)

Dr Gabrielle Becher (West Glasgow Ambulatory Care Hospital)

Dr Giles Dunnill (Bristol Royal Infirmary)

Dr Adam D Ferguson (University Hospitals of Derby and Burton NHS Foundation Trust)

Dr Sharizan Abdul Ghaffar (Ninewells Hospital & Medical School)

Dr John R Ingram (University Hospital of Wales)

Professor Vincent Piguet (University Hospital of Wales)

Dr Svetlana Kavakleiva (Royal Lancaster Infirmary)

Dr Effie Ladoyanni (Russells Hall Hospital)

Dr Joyce A Leman (Queen Margaret Hospital and Victoria Hospital)

Dr Abby E Macbeth (Norfolk and Norwich University Hospitals NHS Foundation Trust) with acknowledgments to Dr Priya Patel, Dr Puran Gurung, David Tomlinson and Joc Keshet-Price (Norfolk and Norwich University Hospitals NHS Foundation Trust)

Dr Areti Makrygeorgou (West Glasgow Ambulatory Care Hospital)

Dr Richard Parslew (Liverpool University Hospitals NHS Foundation Trust)

Dr Andrew Pink (Guy's and St Thomas' NHS Foundation Trust)

Professor Nick Reynolds (Royal Victoria Infirmary, Newcastle upon Tyne NHS Foundation Trust)

Dr Ashish Sharma (Nottingham Circle)

Dr Catriona Sinclair (Broomfield Hospital)

Professor Catherine Smith (Guy's and St Thomas' NHS Foundation Trust)

Dr Roberto Verdolini (The Princess Alexandra Hospital NHS Trust)

Dr Rachel Wachsmuth (Royal Devon and Exeter NHS Foundation Trust)

Dr Marc Wallace (Addenbrooke's Hospital)

Professor Richard Warren (Salford Royal NHS Foundation Trust)

Professor Andrew Wright (Bradford Teaching Hospitals NHS Foundation Trust)

PIC sites and Investigators

Dr Aisling J Ryan (Kings College Hospital)

Dr Anna Chapman and Dr Kavitha Sundararaj (Queen Elizabeth Hospital)

Dr Nisha Arujuna and Dr Abigail Fogo (Kingston Hospital)

Dr Alya Abdul-Wahab, Dr Charlotte Fleming and Dr Ruth Lamb (St George's University Hospitals NHS Foundation Trust)

Dr Jaskiran Azad and Jacqueline Dodds (South Tees Hospitals NHS Foundation Trust)

Sonia Baryschpolec, Dr Hywel Cooper and Dr Alexa R Shipman (Portsmouth Hospitals NHS Trust)

# Additional methods

## Washout periods for double-blind RCT

Table S1: Wash out periods for double-blind RCT

| **Treatment** | **Wash out period** |
| --- | --- |
| Topical treatment that is likely to impact signs and symptoms of psoriasis (e.g. corticosteroids, vitamin D analogues, calcineurin inhibitors, retinoids, keratolytics, tar, urea) | 2 weeks |
| Methotrexate, Ciclosporin, Acitretin, Alitretinoin, Fumaric acid esters | 4 weeks |
| Phototherapy or PUVA | 4 weeks |
| Etanercept or Adalimumab | 4 weeks |
| Infliximab or Ustekinumab or Secukinumab | 3 months |
| Other TNF antagonists | 3 months |
| Other investigational monoclonal antibody | 3 months |
| Other investigational drugs | 30 days or 5 half lives (whichever is longer) |
| Other immunosuppressant / immunomodulatory therapy including intra-articular steroids | 4 weeks or 5 half lives (whichever is longer) |

## Concomitant therapy rules for the double-blind RCT

Table S2: Summary of Concomitant therapy rules for the double-blind RCT

| **Prohibited** | Very potent topical corticosteroids (eg: Dermovate)  Any topical treatment that is likely to impact signs and symptoms of PPP (e.g. corticosteroids, vitamin D analogues, calcineurin inhibitors, retinoids, keratolytics, tar, urea)  Phototherapy or PUVA  Methotrexate, Cyclosporine, Acitretin, Alitretinoin, FAE  Etanercept or Adalimumab  Infliximab or Ustekinumab or Secukinumab  Other TNF antagonists  Other systemic immunosuppressive therapy  Other investigational monoclonal antibody  Other investigational drugs |
| --- | --- |
| **Allowable topical therapy** | Emollients.  Topical hydrocortisone, antihistamine for injection – site reactions  Mild topical corticosteroids for the treatment of psoriasis at sites other than hands and feet, applied with gloves. |
| **Allowable therapy** | Oral antihistamine for injection - site reactions |
| **“Rescue” topical therapy** | Potent corticosteroid od. To be dispensed only by the study team, at the Investigator’s discretion. Amounts prescribed to be recorded. |

## Description of treatment estimands

An estimand is a clear and unambiguous description of a treatment effect that is targeted by an analysis in a clinical trial, reflecting the clinical question posed by the trial objective. In the following we describe the estimands targeted in APRICOT.

**The Primary treatment policy estimand***:* This estimand aims to answer the question ‘What is the treatment effect of anakinra regardless of treatment discontinuation and any other therapies taken?’

For this trial we specifically aim to answer: What is the mean difference in PPPASI (adjusted for baseline PPPASI) after 8 weeks of treatment with anakinra compared to placebo in patients with a confirmed diagnosis of PPP (meeting APRICOT eligibility criteria), regardless of treatment discontinuation for any reason and regardless of initiation of rescue, prohibited or other topical therapy.

Table S3 –Primary estimand attributes

| Estimand attribute | Description |
| --- | --- |
| Population | Patients with confirmed diagnosis of PPP meeting APRICOT eligibility criteria |
| Treatment condition | 8 weeks of treatment with anakinra compared to placebo |
| Outcome variable | PPPASI at week 8 |
| Strategies used to handle Intercurrent events | Study treatment discontinuation – treatment policy^1^  Use of rescue medication – treatment policy^1^  Use of prohibited medication – treatment policy^1^  Use of other topical medication - treatment policy^1^ |
| Population-level summary measure | Mean difference in Week 8 PPPASI (adjusted for baseline) between the anakinra group and the placebo group |

^1^ A treatment policy strategy considers the occurrence of the associated event as irrelevant, and participant data are analysed regardless.

Supplementary estimands on the primary outcome:

1. What is the mean difference in PPPASI (adjusted for baseline PPPASI) after 8 weeks of treatment with anakinra compared to placebo in patients with a confirmed diagnosis of PPP (meeting APRICOT eligibility criteria) **who are able to comply with taking at least 50%** **of daily injections, regardless of initiation of rescue, prohibited or other topical therapy.**
2. What is the mean difference in PPPASI (adjusted for baseline PPPASI) after 8 weeks of treatment with anakinra compared to placebo in patients with a confirmed diagnosis of PPP (meeting APRICOT eligibility criteria) **if rescue therapy was not available**, **and those who initiated on rescue had a worse PPPASI than those in their treatment group not initiated on rescue, regardless of treatment discontinuation, initiation of prohibited or other topical therapy.**
3. What is the mean difference in PPPASI (adjusted for baseline PPPASI) after 8 weeks of treatment with anakinra compared to placebo in patients with a confirmed diagnosis of PPP (meeting APRICOT eligibility criteria) **if rescue and prohibited therapy were not available**, **and those who initiated on rescue or prohibited therapy had a worse PPPASI than those in their treatment group not initiated on rescue or prohibited therapy, regardless of treatment discontinuation** **or other topical therapy.**
4. What is the mean difference in PPPASI (adjusted for baseline PPPASI) after 8 weeks of treatment with anakinra compared to placebo in patients with a confirmed diagnosis of PPP (meeting APRICOT eligibility criteria) **if rescue, prohibited and other topical therapy were not available, and those who initiated on rescue, prohibited or other topical therapy had a worse PPPASI than those in their treatment group not initiated on rescue, prohibited or other topical therapy, regardless of treatment discontinuation.**

Table S4 - Supplementary estimands attributes

| Supplementary estimand | Estimand attribute | Description |
| --- | --- | --- |
| 1. Treatment effect in strata of treatment compliers | Population | The principal strata of patients with confirmed diagnosis of PPP meeting APRICOT eligibility criteria, who would comply with 8 weeks of treatment |
|  | Treatment condition | 8 weeks of treatment with anakinra compared to placebo under compliance^1^ |
|  | Outcome variable | PPPASI at week 8 |
|  | Strategies used to handle Intercurrent events | Study treatment discontinuation – principal stratum^2^  Use of rescue medication – treatment policy^3^  Use of prohibited medication – treatment policy^3^  Use of other topical medication – treatment policy^3^ |
|  | Population-level summary measure | Mean difference in Week 8 PPPASI (adjusted for baseline) |
| 1. Treatment effect if rescue therapy was not available | Population | Patients with confirmed diagnosis of PPP meeting APRICOT eligibility criteria |
|  | Treatment condition | 8 weeks of treatment with anakinra compared to placebo |
|  | Outcome variable | PPPASI at week 8 |
|  | Strategies used to handle Intercurrent events | Study treatment discontinuation – treatment policy^3^  Use of rescue medication – hypothetical^4^  Use of prohibited medication – treatment policy^3^  Use of other topical medication – treatment policy^3^ |
|  | Population-level summary measure | Mean difference in Week 8 PPPASI (adjusted for baseline) between the anakinra group and the placebo group |
| 1. Treatment effect if rescue therapy and prohibited therapy was not available | Population | Patients with confirmed diagnosis of PPP meeting APRICOT eligibility criteria |
|  | Treatment condition | 8 weeks of treatment with anakinra compared to placebo |
|  | Outcome variable | PPPASI at week 8 |
|  | Strategies used to handle Intercurrent events | Study treatment discontinuation – treatment policy^3^  Use of rescue medication – hypothetical^4^  Use of prohibited medication – hypothetical^4^  Use of other topical medication – treatment policy^3^ |
|  | Population-level summary measure | Mean difference in Week 8 PPPASI (adjusted for baseline) between the anakinra group and the placebo group |
| 1. Treatment effect if rescue therapy, prohibited therapy and other topical therapy was not available | Population | Patients with confirmed diagnosis of PPP meeting APRICOT eligibility criteria |
|  | Treatment condition | 8 weeks of treatment with anakinra compared to placebo |
|  | Outcome variable | PPPASI at week 8 |
|  | Strategies used to handle Intercurrent events | Study treatment discontinuation – treatment policy^3^  Use of rescue medication – hypothetical^4^  Use of prohibited medication – hypothetical^4^  Use of other topical medication – hypothetical^4^ |
|  | Population-level summary measure | Mean difference in Week 8 PPPASI (adjusted for baseline) between the anakinra group and the placebo group |

^1^For the main CACE estimand, a complier was defined as a user of 50% or more of the planned injections over the 8 week treatment period. Sensitivity analysis was performed alternatively defining a complier as a user of 60-90% or more of planned injections. ^2^The clinical question of interest relates to the treatment effect only within the **principal stratum** of patients who would comply with treatment. ^3^ A **treatment policy** strategy considers the occurrence of the associated intercurrent event as irrelevant, and participant data are analysed regardless. ^4^ A scenario is envisaged in which the specified event (e.g. rescue medication use) does not occur, and in the absence of the specified event participants had a worse outcome than observed amongst those who did not have the event; for patients who experienced the event their value of the outcome variable is imputed as the value which the variable would have taken in the specified **hypothetical** scenario using Multiple Imputation (See Additional statistical methods for stage 2).

## Additional statistical methods for stage 1

Stage 1 analysis was conducted in accordance with the APRICOT stage 1 statistical analysis plan (SAP) and the intention-to-treat (ITT) principle (1). The baseline adjusted mean treatment group difference in the fresh pustule count and PPASI score, averaged across follow-up visits (week 1-8), was calculated using a linear regression model. The trial continued to stage 2 if the treatment group did better than placebo for at least one measure as assessed by the ordering of mean score (2). The primary outcome for stage 2 was selected from two candidates and based on an assessment of reliability and distributional properties.

At the end of stage 1, two candidate primary outcome were assessed; the fresh pustule count and PPASI score. These were recorded at baseline, and at weeks: 1, 4, 8 and 12 by a blinded assessor at each site. During stage 1 fresh pustule counts were also assessed by a central, blinded assessor using photography (pre-specified views of palms and soles at baseline, week 1 and week 8 of treatment) to enable the reliability of this method of measurement to be assessed. PPPASI was also measured by a second independent assessor at each site to enable the reliability of this outcome to be assessed.

The primary outcome for stage 2 was selected based on the assessment of reliability for each candidate outcome, using the Intraclass Correlation Coefficient (ICC). This was calculated using a mixed effect ANOVA with a random intercept for patient and rater. The method of Bland and Altman was also employed to assess reliability of each outcome measure. Discriminatory ability was assessed using standardised mean differences, and histograms by treatment group. All analyses were conducted using Stata version 15.1. Full details are in the APRICOT stage 1 SAP (1).

## Additional statistical methods for stage 2

The main analysis was based on the intention-to-treat (ITT) principle, that is, all participants with at least one follow-up were analysed in the group to which they were randomised regardless of subsequent treatment received

For the primary outcome, a linear mixed-effects model using data from week 1, 4 and 8, with random intercepts for participant and centre and fixed effects for treatment group × time interaction and baseline PPPASI, estimated the mean between-group difference in PPPASI at 8 weeks with 95% CI and corresponding *P* value. The primary analysis model was fitted using REML and an unstructured covariance structure. Missing responses were assumed to be missing-at-random (MAR).

Pre-planned exploratory analysis included week 12 data in the primary analysis model to assess the treatment effect at week 12.

Planned sensitivity analyses for the primary outcome were performed. Sensitivity analysis targets the same treatment policy estimand as the primary analysis, but under different assumptions for missing data. This included:

Analysis to assess the impact of missing outcome data on the treatment effect using Multiple Imputation (MI) and a pattern mixture approach (3):

- Use of Multiple Imputation (MI) to explore the impact of a worse outcome for participants with missing outcome data. Missing data were imputed assuming a PPPASI score ranging from 0.039 to 0.39 points higher per week unobserved than that predicted under MAR, corresponding to an outcome which was worse by 0-100% of the unadjusted mean weekly change observed in the PPPASI over 8 weeks. The APRICOT SAP pre-specified parameters ranging from 0-50% of the unadjusted mean weekly change, this was extended up to 100% to further test sensitivity. For each MI analysis 50 imputed data sets were generated, the primary analysis model was fitted to each imputed data set and results were combined using Rubin’s rules.

Supplementary analyses for the primary outcome were performed, targeting the 4 estimands outlined in Table S4. These included:

Three sets of analyses to account for concomitant treatment and examine impact on the treatment effect:

- Excluding data post initiation rescue therapy and using MI to explore the impact of a worse outcome post initiation on rescue therapy on trial results. First an assumption of Missing-at-random (MAR) was made for post-rescued data, which provides an estimate of the treatment effect under the assumption that those rescued would have had data similar to those who were not rescued, in the absence of rescue initiation. Subsequently MNAR assumptions that assumed progressively worse outcomes for those rescued, relative to those observed, in the absence of rescue initiation. The primary analysis model was retained for use in the sensitivity analysis, following MI.
- Excluding data post initiation of rescue and prohibited therapy and using MI to explore the impact of a worse outcome post initiation on rescue and prohibited therapy on trial results. First an assumption of Missing-at-random (MAR) was made for post-rescue/prohibited data, which provides an estimate of the treatment effect under the assumption that those rescued or started on prohibited therapy would have had data similar to those who were not rescued or started on prohibited therapy. Subsequently MNAR assumptions that assumed in the absence of rescue or prohibited therapy initiation progressively worse outcomes would have been observed, relative to those observed. The primary analysis model was retained for use in the sensitivity analysis, following MI.
- Excluding data post initiation of topical therapy and using MI to explore the impact of a worse outcome post initiation on topical therapy on trial results. Data during the use of the specified topical treatment was set missing and assumed to be missing-at-random (MAR) conditional on treatment group, baseline PPPASI and observed PPPASI until time of treatment initiation. The primary analysis model was retained for use in the sensitivity analysis, following MI.

Analysis to adjust for non-adherence on the treatment effect:

The complier average causal effect (CACE) was estimated using a two-stage least squares instrumental variable regression for the primary endpoint. This method preserved the benefits of randomisation. Here, we initially defined a ‘complier’ as those who completed more than 50% of planned injections. Randomisation was used as an instrumental variable for treatment received, with the same covariates as in the primary analysis model (excluding centre from the model). We also calculated the CACE where a complier was alternatively defined as receiving 60-90% planned injections.

A post-hoc subgroup analysis was performed to explore whether there was any evidence of an interaction between baseline smoking status (smoker versus none/ex-smoker). The primary analysis model was extended to include the smoking status by treatment by time interaction.

Secondary outcomes:

Linear (Gaussian) mixed regression models were used for the analysis of the continuous secondary outcomes. Binary outcomes were analysed using mixed logistic regression models and ordered categorical outcomes using mixed ordered logistic models. Similar to the primary analysis model, the models for secondary outcomes included participant and centre as a random intercept and fixed effects for time, time-by-treatment group interaction and baseline value of the outcome. Secondary outcome analysis focussed on the treatment effect at week 8, but we also report the treatment effects at weeks 1 and 4. Time to event outcomes were analysed using random-intercept (for centre) complementary log-log modes, given outcomes were observed at relatively few discrete time intervals.

Adverse Events were coded using the Medical Dictionary for Regulatory Activities (MedDRA) and were tabulated by type and summarised visually in a Dot plot to compare absolute and relative differences between treatment group.

All analyses were conducted using Stata version 15.1. Full details are in the APRICOT stage 2 SAP (4).

# Additional results for stage 1

Decision to proceed to Stage 2 and selection of primary outcome

As per protocol, the stage 1 analysis was performed after the randomisation of 24 participants (13 placebo, 11 anakinra between October 2016 to October 2017). The mean treatment group difference in PPPASI was -1.2, 95% CI (-5.5 to 3.1) in favour of anakinra. The mean treatment group difference in the fresh pustule count was higher for anakinra versus placebo 16.5, 95% CI (-51.0 to 49.6). The trial met the criteria to progress to stage 2. The PPPASI was unanimously selected by the independent DMC members as the primary outcome for stage 2, being judged more reliable than fresh pustule count (ICC=0.73 versus ICC=0.13 respectively).

Table S5: Standardised Mean Differences (unadjusted) for stage 1 outcomes

| **Outcome** | **Week** | **Total**  **N** | **Placebo (N)** | **Anakinra (N)** | **SMD (95% CI)** |
| --- | --- | --- | --- | --- | --- |
| Fresh pustule count (site assessed) | Baseline | 23 | 13 | 10 | 0.06 (-0.77, 0.88) |
|  | 1 | 22 | 13 | 9 | -0.18 (-1.03, 0.67) |
|  | 4 | 20 | 12 | 8 | -0.53 (-1.43, 0.39) |
|  | **8*** | **23** | **13** | **10** | **-0.11 (-0.93, 0.72)** |
|  | 12 | 16 | 9 | 7 | -0.04 (-1.02, 0.95) |
| Fresh pustule count (central photographic assessor) | 0 | 24 | 13 | 11 | 0.39 (-0.43, 1.19) |
|  | 1 | 24 | 13 | 11 | 0.37 (-0.45, 1.18) |
|  | **8*** | **22** | **12** | **10** | **0.06 (-0.78, 0.90)** |
| PPPASI (1^st^ site assessor) | Baseline | 24 | 13 | 11 | 0.24 (-0.57, 1.05) |
|  | 1 | 23 | 13 | 10 | -0.25 (-1.07, 0.59) |
|  | 4 | 21 | 12 | 9 | 0.44 (-0.44, 1.31) |
|  | **8*** | **23** | **12** | **11** | **0.41 (-0.42, 1.23)** |
|  | 12 | 17 | 9 | 8 | 0.1 (-0.86, 1.05) |
| PPPASI (2^nd^ site assessor) | 0 | 24 | 13 | 11 | 0.32 (-0.49, 1.12) |
|  | 1 | 23 | 13 | 10 | 0.18 (-0.64, 1.01) |
|  | 4 | 23 | 13 | 10 | 0.48 (-0.36, 1.31) |
|  | **8*** | **24** | **13** | **11** | **0.14 (-0.67, 0.94)** |
|  | 12 | 17 | 9 | 8 | 0.08 (-0.87, 1.04) |

SMD > 0 favours anakinra. SMDs are unadjusted estimates.*Primary endpoint time.

Figure S1 Agreement between site assessor and photographic central assessment for fresh pustule count

Figure S2 Agreement between site assessor 1 and 2 for PPPASI

# Additional results for stage 2

Table S6 Self-reported adherence to treatment (including treatment withdrawals for overall adherence)

| **Treatment period (n=64):** | **Number of doses per week**  **(mean of SMS and self-reported)** | | | |
| --- | --- | --- | --- | --- |
|  | **Placebo (N=33)*** | | **Anakinra (N=31)‡** | |
|  | **N** | **Mean (SD)** | **N** | **Mean (SD)** |
| Week 1 | 30 | 6.1 (1.9) | 29 | 6.7 (0.6) |
| Week 2 | 30 | 5.9 (2.2) | 29 | 6.7 (0.8) |
| Week 3 | 30 | 6.2 (1.9) | 29 | 5.9 (2.1) |
| Week 4 | 30 | 6.2 (2.1) | 29 | 5.9 (2.1) |
| Week 5 | 30 | 5.7 (2.6) | 29 | 5.7 (2.5) |
| Week 6 | 31 | 5.1 (2.9) | 29 | 5.3 (2.7) |
| Week 7 | 31 | 4.9 (3.1) | 29 | 5.5 (2.6) |
| Week 8 | 31 | 4.8 (3.1) | 29 | 5.3 (2.7) |

*2 in Placebo missing all adherence data and a third with partial adherence data. 2 in Anakinra also missing adherence data ^1^Use of medication confirmed daily from a SMS response of ‘yes’. ^2^Use of medication self-reported at each clinic visit for each day since the previous visit in response to the question ‘Injection taken? Permanent and Temporary treatment discontinuations are included as 0 doses per week at the associated time points.

Table S7 Proportions of compliers for ≥50 to ≥90% total planned injections received

| **Compliance†** | **Placebo** | **Anakinra** |
| --- | --- | --- |
| ≥50% injections |  |  |
| Yes | 26 (79%) | 25 (81%) |
| No | 7 (21%) | 6 (19%) |
| ≥60% injections |  |  |
| Yes | 24 (73%) | 24 (77%) |
| No | 9 (27%) | 7 (23%) |
| ≥70% injections |  |  |
| Yes | 22 (67%) | 24 (77%) |
| No | 11 (33%) | 7 (23%) |
| ≥80% injections |  |  |
| Yes | 22 (67%) | 23 (74%) |
| No | 11 (33%) | 8 (26%) |
| ≥90% injections |  |  |
| Yes | 20 (61%) | 15 (48%) |
| No | 13 (39%) | 16 (52%) |

† All individuals missing compliance data are assumed to be non-compliant in accordance with the APRICOT SAP.

S8 Rescue therapy use over 12 week follow-up by treatment group

| **Treatment given (N, %)** | **Placebo**  **N=33** | **Anakinra**  **N=31** | **Total**  **N=64** |
| --- | --- | --- | --- |
| Moderately potent corticosteroid |  |  |  |
| Clobetasone butyrate | 1 (3%) | 0 (0%) | 1 (2%) |
| Potent corticosteroid |  |  |  |
| Betamethasone valerate† | 5 (15%) | 5 (16%) | 10 (16%) |
| Betamethasone dipropionate and salicylic acid (Diprosalic ^TM^) | 1 (3%) | 0 (0%) | 1 (2%) |
| Mometasone furoate | 5 (15%) | 8 (26%) | 13 (20%) |
| **Total number of participants** | **11 (33%)** | **12 (39%)** | **23 (36%)** |

One participant in the placebo group and one participant in the anakinra group were initiated on two different types of rescue medication during follow-up.

†One participant in the placebo group and one participant in the anakinra group were initiated on betamethasone valerate in the form of Fucibet^TM^.

S9 Time of first initiation on rescue therapy by treatment group

| **Rescue initiation (N, %)** | **Placebo**  **N=33** | **Anakinra**  **N=31** | **Total**  **N=64** |
| --- | --- | --- | --- |
| Baseline | 0 (0%) | 2 (6%) | 2 (3%) |
| Prior to week 1 visit | 0 (0%) | 0 (0%) | 0 (0%) |
| Prior to week 4 visit | 3 (9%) | 8 (26%) | 11 (17%) |
| Prior to week 8 visit | 5 (15%) | 1 (3%) | 6 (9%) |
| Prior to week 12 visit | 3 (9%) | 1 (3%) | 4 (6%) |
| **Total (n, %)** | **11 (33%)** | **12 (39%)** | **23 (36%)** |

S10 Prohibited therapy use over 12 week follow-up by treatment group

| **Treatment given (N, %)** | **Placebo**  **N=33** | **Anakinra**  **N=31** | **Total**  **N=64** |
| --- | --- | --- | --- |
| Topical super-potent corticosteroid |  |  |  |
| Clobetasol propionate | 3 (9%) | 4 (13%) | 7 (11%) |
| Systemic therapy |  |  |  |
| Acitretin | 2 (6%) | 2 (6%) | 4 (6%) |
| Ciclosporin | 0 (0%) | 1 (3%) | 1 (2%) |
| Prednisolone | 1 (3%) | 0 (0%) | 1 (2%) |
| **Total No. of participants†** | **5 (15%)** | **5 (16%)** | **10 (22%)** |

†1 participant in the placebo group and 2 in the anakinra group received more than one prohibited treatment during trial follow-up.

S11 Time point of first initiation on prohibited therapy by treatment group

| **Prohibited initiation (N, %)** | **Placebo**  **N=33** | **Anakinra**  **N=31** | **Total**  **N=64** |
| --- | --- | --- | --- |
| Baseline | 0 (0%) | 0 (0%) | 0 (0%) |
| Prior to week 1 visit | 0 (0%) | 0 (0%) | 0 (0%) |
| Prior to week 4 visit | 0 (0%) | 0 (0%) | 0 (0%) |
| Prior to week 8 visit | 3 (9%)† | 3 (10%)‡ | 6 (9%) |
| Prior to week 12 visit | 2 (6%) | 2 (6%) | 4 (6%) |
| **Total** | **5 (15%)** | **5 (16%)** | **10 (22%)** |

†Two participants started on prohibited therapy on the week 4 visit date and 1 started the day after the week 4 visit date. ‡Two participants started on the week 4 visit data and 1 started in week 5. All participants initiating on prohibited therapy were withdrawn from trial treatment as per APRICOT protocol.

S12 Other topical treatment use over 12 week follow-up by treatment group (excluding topical rescue and prohibited treatments)

| **Treatment given (N, %)** | **Placebo**  **N=33** | **Anakinra**  **N=31** | **Total**  **N=64** |
| --- | --- | --- | --- |
| Antiseptics, antibiotics or antifungals |  |  |  |
| Naseptin | 1 (3%) | 0 (0%) | 1 (2%) |
| Nystatin | 0 (0%) | 1 (3%) | 1 (2%) |
| Mild corticosteroids |  |  |  |
| Hydrocortisone | 1 (3%) | 7 (23%) | 8 (13%) |
| Clobetasone 17- butyrate (with oxytetracycline & nystatin [Trimovate ^TM^]) | 0 (0%) | 1 (3%) | 1 (2%) |
| Potent corticosteroids |  |  |  |
| Betamethasone valerate | 1 (3%) | 2 (6%) | 3 (5%) |
| Emollients |  |  |  |
| Cetraban | 1 (3%) | 1 (3%) | 2 (3%) |
| Dermol | 1 (3%) | 1 (3%) | 2 (3%) |
| Doublebase | 0 (0%) | 1 (3%) | 1 (2%) |
| E45 | 1 (1%) | 0 (0%) | 1 (2%) |
| Epaderm | 3 (9%) | 2 (6%) | 5 (8%) |
| **Total number of participants†** | **8 (24%)** | **13 (42%)** | **21 (33%)** |

†1 participant in Placebo and 3 in Anakinra received more than one other topical treatment during trial follow-up.

S13 Time point of first initiation of other topical therapy by treatment group (excluding topical rescue and prohibited treatments)

| **Rescue initiation (N, %)** | **Placebo**  **N=33** | **Anakinra**  **N=31** | **Total**  **N=64** |
| --- | --- | --- | --- |
| Baseline | 1 (3%) | 1 (3%) | 2 (3%) |
| Prior to week 1 visit | 0 (0%) | 1 (3%) | 1 (2%) |
| Prior to week 4 visit | 4 (12%) | 8 (26%) | 12 (19%) |
| Prior to week 8 visit | 2 (6%) | 2 (6%) | 5 (8%) |
| Prior to week 12 visit | 1 (3%) | 1 (3%) | 2 (3%) |
| **Total** | **8 (24%)** | **13 (42%)** | **22 (34%)** |

Table S14 Sensitivity analysis exploring the impact of missing data

| **Analysis** | **Mean treatment group difference in week 8 PPPASI:**  **A - P [95%CI]** | **P value** |
| --- | --- | --- |
| **Primary analysis** |  |  |
| **MAR using Mixed Model (N=62)** | **-1.65 [-4.77 to 1.47]** | **0.300** |
| MAR using MI (N=64) | -1.66 [-5.11 to 1.79] | 0.346 |
| **MNAR‡ sensitivity analysis (N=64)** |  |  |
| MNAR using MI – MAR + 10%*δ | -1.66 [-5.11 to 1.80] | 0.347 |
| MNAR using MI – MAR + 20%*δ | -1.66 [-5.11 to 1.80] | 0.348 |
| MNAR using MI – MAR + 30%*δ | -1.65 [-5.11 to 1.80] | 0.348 |
| MNAR using MI – MAR + 40%*δ | -1.65 [-5.11 to 1.81] | 0.349 |
| MNAR using MI – MAR + 50%*δ | -1.65 [-5.11 to 1.81] | 0.350 |
| MNAR using MI – MAR + 60%*δ | -1.65 [-5.11 to 1.81] | 0.350 |
| MNAR using MI – MAR + 70%*δ | -1.65 [-5.11 to 1.82] | 0.351 |
| MNAR using MI – MAR + 80%*δ | -1.65 [-5.11 to 1.82] | 0.352 |
| MNAR using MI – MAR + 90%*δ | -1.65 [-5.11 to 1.82] | 0.352 |
| MNAR using MI – MAR + 100%*δ | -1.64 [-5.11 to 1.83] | 0.353 |
|  |  |  |
| **MNAR‡ in Placebo (N=64)** |  |  |
| MNAR using MI – MAR + 10%*δ | -1.67 [-5.13 to 1.78] | 0.343 |
| MNAR using MI – MAR + 20%*δ | -1.69 [-5.14 to 1.77] | 0.339 |
| MNAR using MI – MAR + 30%*δ | -1.70 [-5.16 to 1.76] | 0.335 |
| MNAR using MI – MAR + 40%*δ | -1.71 [-5.17 to 1.74] | 0.331 |
| MNAR using MI – MAR + 50%*δ | -1.73 [-5.19 to 1.73] | 0.328 |
| MNAR using MI – MAR + 60%*δ | -1.74 [-5.20 to 1.72] | 0.324 |
| MNAR using MI – MAR + 70%*δ | -1.75 [-5.22 to 1.71] | 0.321 |
| MNAR using MI – MAR + 80%*δ | -1.77 [-5.23 to 1.70] | 0.318 |
| MNAR using MI – MAR + 90%*δ | -1.78 [-5.25 to 1.69] | 0.314 |
| MNAR using MI – MAR + 100%*δ | -1.78 [-5.26 to 1.70] | 0.315 |
|  |  |  |
| **MNAR‡ in Anakinra (N=64)** |  |  |
| MNAR using MI – MAR + 10%*δ | -1.64 [-5.10 to 1.81] | 0.351 |
| MNAR using MI – MAR + 20%*δ | -1.63 [-5.08 to 1.82] | 0.355 |
| MNAR using MI – MAR + 30%*δ | -1.61 [-5.07 to 1.84] | 0.360 |
| MNAR using MI – MAR + 40%*δ | -1.60 [-5.05 to 1.86] | 0.365 |
| MNAR using MI – MAR + 50%*δ | -1.58 [-5.04 to 1.87] | 0.369 |
| MNAR using MI – MAR + 60%*δ | -1.57 [-5.02 to 1.89] | 0.374 |
| MNAR using MI – MAR + 70%*δ | -1.55 [-5.01 to 1.90] | 0.378 |
| MNAR using MI – MAR + 80%*δ | -1.54 [-4.99 to 1.92] | 0.382 |
| MNAR using MI – MAR + 90%*δ | -1.52 [-4.98 to 1.93] | 0.387 |
| MNAR using MI – MAR + 100%*δ | -1.51 [-4.96 to 1.95] | 0.392 |

**‡**Missing data were imputed assuming a PPPASI score ranging from 0.039 to 0.39 points higher per week unobserved than that predicted under MAR, corresponding to an outcome which was worse by 0-100% of the unadjusted mean weekly change observed in the PPPASI over 8 weeks. The APRICOT SAP pre-specified parameters ranging from 0-50% of the unadjusted mean weekly change, this was extended up to 100% to further test sensitivity. For each MI analysis 50 imputed data sets were generated, the primary analysis model was fitted to each imputed data set and results were combined using Rubin’s rules.

Table S15 Treatment effect in the absence of rescue therapy use

| **Analysis** | **Mean treatment group difference in week 8 PPPASI:**  **A - P [95%CI]** | **P value** |
| --- | --- | --- |
| **MAR** |  |  |
| MAR using Mixed Model (N=59) | **-2.57 [-6.22 to 1.08]** | 0.167 |
| MAR using MI (N=64) | **-2.30 [-7.48 to 2.87]** | 0.381 |
|  |  |  |
| **MNAR (N=64)** |  |  |
| MNAR using MI – MAR + 10%*δ | -2.26 [-7.43 to 2.92] | 0.390 |
| MNAR using MI – MAR + 20%*δ | -2.21 [-7.39 to 2.96] | 0.400 |
| MNAR using MI – MAR + 30%*δ | -2.17 [-7.34 to 3.10] | 0.410 |
| MNAR using MI – MAR + 40%*δ | -2.12 [-7.29 to 3.06] | 0.422 |
| MNAR using MI – MAR + 50%*δ | -2.07 [-7.25 to 3.11] | 0.431 |

Table S16 Treatment effect in the absence of rescue and prohibited therapy

| **Analysis** | **Mean treatment group difference in week 8 PPPASI:**  **A - P [95%CI]** | **P value** |
| --- | --- | --- |
| **MAR** |  |  |
| MAR using Mixed Model (N=59) | -1.86 [-5.41 to 1.69] | 0.303 |
| MAR using MI (N=64) | -2.09 [-8.47 to 4.29] | 0.518 |
|  |  |  |
| **MNAR (N=64)** |  |  |
| MNAR using MI – MAR + 10%*δ | -2.04 [-8.42 to 4.35] | 0.528 |
| MNAR using MI – MAR + 20%*δ | -1.99 [-8.37 to 4.40] | 0.539 |
| MNAR using MI – MAR + 30%*δ | -1.93 [-8.32 to 4.46] | 0.551 |
| MNAR using MI – MAR + 40%*δ | -1.88 [-8.27 to 4.51] | 0.562 |
| MNAR using MI – MAR + 50%*δ | -1.83 [-8.22 to 4.56] | 0.572 |

Table S17 Treatment effect in the absence of topical therapy

| **Supplementary analysis** | **Mean treatment group difference in week 8 PPPASI:**  **A - P [95%CI]** | **P value** |
| --- | --- | --- |
| 1) Data during topical treatment (rescue/prohibited or other topical) set missing and assumed to be MAR [where missing stop date assumed topical use ongoing] | 0.30 [-3.24 to 3.85] | 0.866 |
| 2) Data during topical treatment (rescue/prohibited or other topical) set missing and assumed to be MAR [where missing stop date assume topical use at closest visit only] | -0.47 [-3.77 to 2.82] | 0.779 |
| 3) Data post rescue/prohibited initiation and only during other topical treatment set missing [where missing stop date assumed other topical use ongoing] | 0.08 [-3.64 to 3.80] | 0.967 |
| 4) Data post rescue/prohibited initiation and only during other topical treatment set missing [where missing stop date assumed topical use at closest visit only] | -1.02 [-4.63 to 2.59] | 0.580 |

Supplementary estimands 1-4 described in Table S4.

Table S18: Treatment acceptability

| **Found the treatment worthwhile** | **Placebo** | **Anakinra** |
| --- | --- | --- |
| Strongly agree | 4 (14%) | 12 (41%) |
| Agree | 8 (29%) | 7 (24%) |
| Neither agree or disagree | 7 (25%) | 6 (21%) |
| Disagree | 5 (18%) | 3 (10%) |
| Strongly disagree | 4 (14%) | 1 (3%) |
| Total | 28 | 29 |

Table S19 Bloods

|  | **Placebo** | | | **Anakinra** | | |  |  |  |  |
| --- | --- | --- | --- | --- | --- | --- | --- | --- | --- | --- |
| **Time** | **N** | **Mean** | **SD** | **N** | **Mean** | **SD** |  | **Unadjusted Mean Difference: A-P**  **(95% CI)** | **Adjusted† Mean Difference: A-P (95% CI)** | **P-value** |
| **Neutrophil Count (x10^9/L)** | | | | | | | | |  |  |
| Baseline | 32 | 5.0 | 1.7 | 29 | 4.9 | 1.5 |  |  |  |  |
| Week 8 | 30 | 5.1 | 1.7 | 31 | 4.3 | 2.3 |  | -0.8 (-1.8, 0.3) |  |  |
| Week 8 change | 30 | 0.2 | 1.3 | 29 | -0.7 | 2.1 |  | -0.9 (-1.8, 0.0) | -0.9 (-1.7, 0.01) | 0.053 |
| **Total White Cell Count (x10^9/L)** | | | | | | | | |  |  |
| Baseline | 32 | 8.3 | 2.0 | 29 | 8.0 | 2.1 |  |  |  |  |
| Week 8 | 30 | 8.4 | 2.6 | 31 | 7.4 | 2.6 |  | -1.0 (-2.3, 0.3) |  |  |
| Week 8 change | 30 | 0.3 | 1.7 | 29 | -0.7 | 2.2 |  | -1.0 (-2.0, 0.1) | -1.0 (-2.01, 0.00) | 0.051 |
| **Haemoglobin (g/L)** | | | | | | | | |  |  |
| Baseline | 32 | 139.7 | 8.9 | 29 | 137.7 | 10.3 |  |  |  |  |
| Week 8 | 30 | 138.4 | 7.1 | 31 | 140.5 | 9.2 |  | 2.1 (-2.2, 6.3) |  |  |
| Week 8 change | 30 | -0.1 | 5.1 | 29 | 2.2 | 6.4 |  | 2.3 (-0.7, 5.3) | 2.0 (-0.6, 4.7) | 0.129 |
| **Platelets (x10^9/L)** | | | | | | | | |  |  |
| Baseline | 32 | 283.8 | 74.3 | 29 | 272.3 | 65.6 |  |  |  |  |
| Week 8 | 30 | 279.9 | 68.5 | 31 | 254.1 | 57.8 |  | -25.8 (-58.2, 6.7) |  |  |
| Week 8 change | 30 | 2.0 | 27.8 | 29 | -22.2 | 33.8 |  | -24.2 (-40.3, -8.1) | -25.3 (-39.6, -11.1) | <0.001 |
| **CRP (mg/L)** | | | | | | | | |  |  |
| Baseline | 26 | 5.0 | 5.7 | 27 | 6.2 | 7.6 |  |  |  |  |
| Week 8 | 9 | 3.2 | 2.4 | 8 | 3.1 | 2.2 |  | -0.1 (-2.5, 2.3) |  |  |
| Week 8 change | 9 | -1.4 | 2.0 | 7 | 0.0 | 0.6 |  | 1.4 (-0.2, 3.1) | 1.05 (-0.5, 2.6) | 0.174 |

**†**Adjusted for baseline blood value and centre using linear mixed model.

Table S20 Adverse events and reactions at preferred term by treatment group

| **AE term** | **Placebo**  **N events** | **Anakinra**  **N events** | **Total**  **N events** | **Placebo**  **N partic.** | **Anakinra**  **N partic.** | **Total**  **N partic.** |
| --- | --- | --- | --- | --- | --- | --- |
| Abdominal discomfort | 1 | 0 | 1 | 1 | 0 | 1 |
| Abdominal pain lower | 0 | 1 | 1 | 0 | 1 | 1 |
| Arthralgia | 2 | 1 | 3 | 2 | 1 | 3 |
| Back injury | 1 | 0 | 1 | 1 | 0 | 1 |
| Biopsy skin | 0 | 1 | 1 | 0 | 1 | 1 |
| Blood creatinine increased | 1 | 0 | 1 | 1 | 0 | 1 |
| Blood pressure increased | 0 | 1 | 1 | 0 | 1 | 1 |
| C-reactive protein increased | 1 | 1 | 2 | 1 | 1 | 2 |
| Catarrh | 1 | 0 | 1 | 1 | 0 | 1 |
| Cellulitis | 1 | 0 | 1 | 1 | 0 | 1 |
| Constipation | 0 | 1 | 1 | 0 | 1 | 1 |
| Contusion | 2 | 1 | 3 | 2 | 1 | 3 |
| Cough | 2 | 5 | 7 | 2 | 4 | 6 |
| Cystitis | 1 | 0 | 1 | 1 | 0 | 1 |
| DNA antibody positive | 1 | 0 | 1 | 1 | 0 | 1 |
| Decreased appetite | 1 | 0 | 1 | 1 | 0 | 1 |
| Depressed mood | 0 | 3 | 3 | 0 | 3 | 3 |
| Dermatitis | 1 | 0 | 1 | 1 | 0 | 1 |
| Diabetes mellitus | 1 | 0 | 1 | 1 | 0 | 1 |
| Diarrhoea | 0 | 5 | 5 | 0 | 5 | 5 |
| Dizziness | 0 | 1 | 1 | 0 | 1 | 1 |
| Ear pain | 1 | 0 | 1 | 1 | 0 | 1 |
| Eosinophilia | 0 | 1 | 1 | 0 | 1 | 1 |
| Epistaxis | 2 | 0 | 2 | 1 | 0 | 1 |
| Flushing | 1 | 0 | 1 | 1 | 0 | 1 |
| Folliculitis | 1 | 2 | 3 | 1 | 1 | 2 |
| Gestational diabetes | 0 | 1 | 1 | 0 | 1 | 1 |
| Glomerular filtration rate decreased | 1 | 0 | 1 | 1 | 0 | 1 |
| Glucose urine present | 1 | 0 | 1 | 1 | 0 | 1 |
| Haematuria | 1 | 2 | 3 | 1 | 2 | 3 |
| Head injury | 0 | 1 | 1 | 0 | 1 | 1 |
| Headache | 4 | 6 | 10 | 2 | 6 | 8 |
| Hepatitis B antibody positive | 0 | 1 | 1 | 0 | 1 | 1 |
| Hepatotoxicity | 1 | 4 | 5 | 1 | 4 | 5 |
| Hyperkalaemia | 1 | 0 | 1 | 1 | 0 | 1 |
| Hypertension | 0 | 1 | 1 | 0 | 1 | 1 |
| Influenza | 1 | 0 | 1 | 1 | 0 | 1 |
| Influenza like illness | 1 | 0 | 1 | 1 | 0 | 1 |
| Injection site discomfort | 0 | 1 | 1 | 0 | 1 | 1 |
| Injection site erythema | 1 | 2 | 3 | 1 | 2 | 3 |
| Injection site pain | 0 | 1 | 1 | 0 | 1 | 1 |
| Injection site pruritus | 1 | 0 | 1 | 1 | 0 | 1 |
| Injection site rash | 0 | 1 | 1 | 0 | 1 | 1 |
| Injection site reaction | 1 | 20 | 21 | 1 | 19 | 20 |
| Injection site swelling | 1 | 2 | 3 | 1 | 2 | 3 |
| Lethargy | 1 | 0 | 1 | 1 | 0 | 1 |
| Lower respiratory tract infection | 3 | 3 | 6 | 3 | 3 | 6 |
| Lymphadenopathy | 1 | 0 | 1 | 1 | 0 | 1 |
| Malaise | 2 | 0 | 2 | 1 | 0 | 1 |
| Mean cell volume increased | 1 | 0 | 1 | 1 | 0 | 1 |
| Menorrhagia | 0 | 1 | 1 | 0 | 1 | 1 |
| Metrorrhagia | 0 | 1 | 1 | 0 | 1 | 1 |
| Migraine | 2 | 0 | 2 | 2 | 0 | 2 |
| Monocyte count increased | 1 | 0 | 1 | 1 | 0 | 1 |
| Myalgia | 1 | 0 | 1 | 1 | 0 | 1 |
| Nasopharyngitis | 3 | 5 | 8 | 3 | 4 | 7 |
| Nausea | 2 | 2 | 4 | 2 | 2 | 4 |
| Neuralgia | 0 | 1 | 1 | 0 | 1 | 1 |
| Neutrophil count increased | 0 | 1 | 1 | 0 | 1 | 1 |
| Oedema peripheral | 0 | 1 | 1 | 0 | 1 | 1 |
| Oropharyngeal pain | 1 | 3 | 4 | 1 | 3 | 4 |
| Osteoporosis | 1 | 0 | 1 | 1 | 0 | 1 |
| Pain in extremity | 1 | 1 | 2 | 1 | 1 | 2 |
| Pain of skin | 0 | 1 | 1 | 0 | 1 | 1 |
| Pharyngeal oedema | 1 | 0 | 1 | 1 | 0 | 1 |
| Post procedural infection | 1 | 0 | 1 | 1 | 0 | 1 |
| Pregnancy | 0 | 1 | 1 | 0 | 1 | 1 |
| Proteinuria | 0 | 1 | 1 | 0 | 1 | 1 |
| Pruritus | 0 | 1 | 1 | 0 | 1 | 1 |
| Psoriasis | 2 | 3 | 5 | 2 | 3 | 5 |
| Psoriatic arthropathy | 1 | 0 | 1 | 1 | 0 | 1 |
| Pustular psoriasis | 2 | 2 | 4 | 2 | 2 | 4 |
| Pyuria | 0 | 1 | 1 | 0 | 1 | 1 |
| Rash macular | 1 | 0 | 1 | 1 | 0 | 1 |
| Rash papular | 1 | 0 | 1 | 1 | 0 | 1 |
| Rhinitis | 1 | 1 | 2 | 1 | 1 | 2 |
| Rhinitis allergic | 0 | 1 | 1 | 0 | 1 | 1 |
| Rhinorrhoea | 0 | 1 | 1 | 0 | 1 | 1 |
| Sinusitis | 1 | 2 | 3 | 1 | 2 | 3 |
| Skin infection | 2 | 1 | 3 | 2 | 1 | 3 |
| Skin irritation | 1 | 0 | 1 | 1 | 0 | 1 |
| Skin lesion | 1 | 0 | 1 | 1 | 0 | 1 |
| Synovial cyst | 1 | 0 | 1 | 1 | 0 | 1 |
| Synovitis | 1 | 0 | 1 | 1 | 0 | 1 |
| Tonsillitis | 0 | 1 | 1 | 0 | 1 | 1 |
| Toothache | 1 | 0 | 1 | 1 | 0 | 1 |
| Transaminases increased | 0 | 1 | 1 | 0 | 1 | 1 |
| Upper respiratory tract infection | 0 | 1 | 1 | 0 | 1 | 1 |
| Urinary tract infection | 3 | 4 | 7 | 3 | 4 | 7 |
| Urine analysis abnormal | 0 | 1 | 1 | 0 | 1 | 1 |
| Viral infection | 2 | 0 | 2 | 2 | 0 | 2 |
| Visual acuity reduced | 1 | 0 | 1 | 1 | 0 | 1 |
| Vomiting | 0 | 2 | 2 | 0 | 2 | 2 |
| White blood cell count increased | 0 | 1 | 1 | 0 | 1 | 1 |
| White blood cells urine positive | 2 | 0 | 2 | 2 | 0 | 2 |
| Urine analysis abnormal | 1 | 1 | 2 | 1 | 1 | 2 |

Post-hoc exploratory analysis exploring association between smoking status and treatment effect:

There was no evidence that the treatment effect on PPPASI differed by smoking status. A test of the interaction between smoking status and treatment effect in PPPASI at week 8 was not significant (p=0.925, interaction effect=-0.31, 95% CI -6.69 to 6.08).

Table S21 – PPPASI by treatment group and smoking status

| Status | Mean 8 week change in PPPASI, 95% CI | |
| --- | --- | --- |
|  | Placebo | Anakinra |
| Smoker | -1.1 95% CI[-5.8 to 3.6] | -3.9 95% CI[-8.7 to 0.8] |
| Non-smoker | -3.4 95% CI [-7.9 to 1.1] | -4.5 95% CI [-7.9 to 1.0] |

Note: confidence intervals largely overall for these 4 groups

# Minimum Clinically Important Difference

The Minimum Clinically Important Difference (MCID) is the smallest difference in an outcome measure that represents a clinically relevant outcome to the patient, regardless of cost and burden. Published studies have recommended the use of both anchor- and distribution-based methods to determine the MCID (5, 6).

One distribution-based method involves estimating the MCID as a fraction of the Standard deviation (SD). A systematic review reported convergence upon a value of 0.5 SD for the MCID in health-related quality of life for chronic diseases (7). The SD of the baseline PPPASI was calculated for the 64 randomised patients. The SD in week 8 change from baseline, removing the treatment effect, was calculated as the root mean squared error resulting from a one-way ANOVA comparing change in baseline across treatment groups (6).

Distribution based approach:

Baseline SD in PPPASI was 10.5 (N=64). ½ SD = 5.25

Change in PPPASI from baseline to week 8, SD = 8.08 (N=59). ½ SD = 4.04

Adopting a distribution based approach results suggest a crude estimate of the MCID ranging between 4 to 5.25.

Anchor based approach:

Anchor based methods entail comparing the week 8 change in the PPPASI with a second measure of change, which serves as the anchor. The mean week 8 change in PPPASI was examined for a change in PPP IGA from severe to moderate, moderate to mild and a two point change from moderate to almost clear (no other combinations were available in the data) by treatment group and overall (table S22). These results should be interpreted with due caution due to the limited sample size and treatment group difference, however indicate a MCID between 4 to 5.25 (across all randomised participants) is consistent with a change in PPP IGA from severe to moderate, moderate to milk and moderate to almost clear.

Table S22 – Exploring MCID using Anchor based approach

| Anchor | placebo  Mean change in PPPASI | placebo N | anakinra  Mean change in PPPASI | anakinra  N | Total (anakinra + placebo) Mean change in PPPASI | N |
| --- | --- | --- | --- | --- | --- | --- |
| PPP IGA severe to moderate | 2.25 | 4 | -10.9 | 5 | -5.06 | 9 |
| PPP IGA moderate to mild | -3.6 | 2 | -3.5 | 3 | -3.5 | 5 |
| PPP IGA moderate to almost clear | -4.3 | 2 | -5.4 | 1 | -4.7 | 3 |

**References**

1. Cro S, Smith C, Wilson R, Cornelius V. Treatment of pustular psoriasis with anakinra: a statistical analysis plan for stage 1 of an adaptive two-staged randomised placebo-controlled trial. Trials. 2018;19(1):534.

2. Piantadosi S. Clinical Trials: A Methodologic Perspective. Edition 2 ed: Wiley; 2017.

3. Cro S, Morris TP, Kenward MG, Carpenter JR. Sensitivity analysis for clinical trials with missing continuous outcome data using controlled multiple imputation: A practical guide. Statistics in Medicine. 2020;39(21):2815-42.

4. Cro S, Patel P, Barker J, Burden DA, Griffiths CEM, Lachmann HJ, et al. A randomised placebo controlled trial of anakinra for treating pustular psoriasis: statistical analysis plan for stage two of the APRICOT trial. Trials. 2020;21(1):158-.

5. Rai SK, Yazdany J, Fortin PR, Aviña-Zubieta JA. Approaches for estimating minimal clinically important differences in systemic lupus erythematosus. Arthritis Res Ther. 2015;17(1):143-.

6. Howard R, Phillips P, Johnson T, O'Brien J, Sheehan B, Lindesay J, et al. Determining the minimum clinically important differences for outcomes in the DOMINO trial. International Journal of Geriatric Psychiatry. 2011;26(8):812-7.

7. Norman GR, Sloan JA, Wyrwich KW. Interpretation of changes in health-related quality of life: the remarkable universality of half a standard deviation. Medical care. 2003;41(5):582-92.
