# Supplementary material for: Anakinra for palmoplantar pustulosis: results from a randomized, double‐blind, multicentre, two‐staged, adaptive placebo‐controlled trial (APRICOT)
Source: Br J Dermatol. 2021 Oct 12;186(2):245–56. doi: 10.1111/bjd.20653 (PMC9255857; doi:10.1111/bjd.20653)
Supplement: Supplementary file 4 — Powerpoint S1 Journal Club Slide Set. [file BJD-186-245-s004.pptx]

## Slide 1
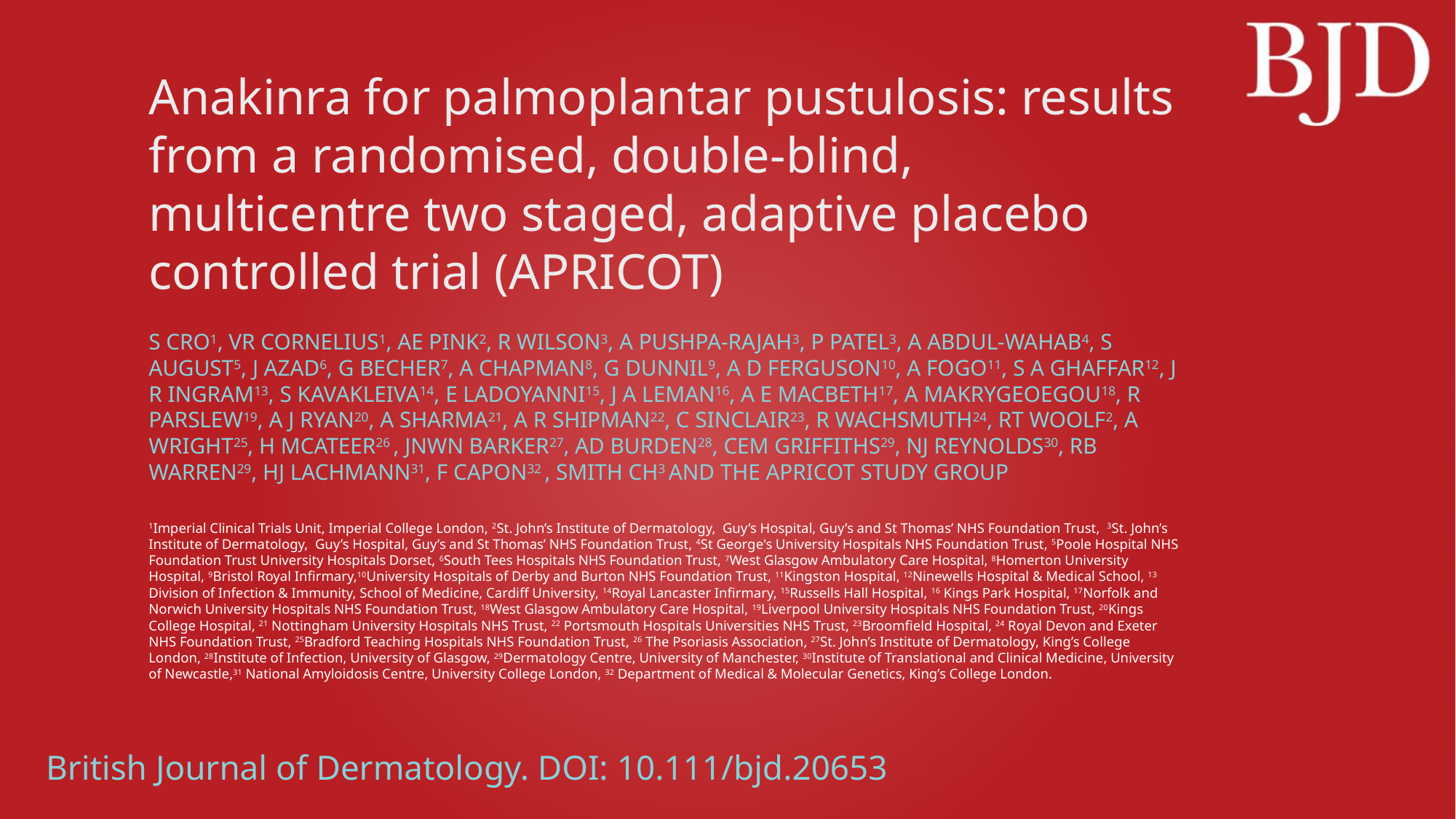

# Anakinra for palmoplantar pustulosis: results from a randomised, double-blind, multicentre two staged, adaptive placebo controlled trial (APRICOT)
S CRO1, VR CORNELIUS1, AE PINK2, R WILSON3, A PUSHPA-RAJAH3, P PATEL3, A ABDUL-WAHAB4, S AUGUST5, J AZAD6, G BECHER7, A CHAPMAN8, G DUNNIL9, A D FERGUSON10, A FOGO11, S A GHAFFAR12, J R INGRAM13, S KAVAKLEIVA14, E LADOYANNI15, J A LEMAN16, A E MACBETH17, A MAKRYGEOEGOU18, R PARSLEW19, A J RYAN20, A SHARMA21, A R SHIPMAN22, C SINCLAIR23, R WACHSMUTH24, RT WOOLF2, A WRIGHT25, H MCATEER26 , JNWN BARKER27, AD BURDEN28, CEM GRIFFITHS29, NJ REYNOLDS30, RB WARREN29, HJ LACHMANN31, F CAPON32 , SMITH CH3 AND THE APRICOT STUDY GROUP
1Imperial Clinical Trials Unit, Imperial College London, 2St. John’s Institute of Dermatology, Guy’s Hospital, Guy’s and St Thomas’ NHS Foundation Trust, 3St. John’s Institute of Dermatology, Guy’s Hospital, Guy’s and St Thomas’ NHS Foundation Trust, 4St George's University Hospitals NHS Foundation Trust, 5Poole Hospital NHS Foundation Trust University Hospitals Dorset, 6South Tees Hospitals NHS Foundation Trust, 7West Glasgow Ambulatory Care Hospital, 8Homerton University Hospital, 9Bristol Royal Infirmary,10University Hospitals of Derby and Burton NHS Foundation Trust, 11Kingston Hospital, 12Ninewells Hospital & Medical School, 13 Division of Infection & Immunity, School of Medicine, Cardiff University, 14Royal Lancaster Infirmary, 15Russells Hall Hospital, 16 Kings Park Hospital, 17Norfolk and Norwich University Hospitals NHS Foundation Trust, 18West Glasgow Ambulatory Care Hospital, 19Liverpool University Hospitals NHS Foundation Trust, 20Kings College Hospital, 21 Nottingham University Hospitals NHS Trust, 22 Portsmouth Hospitals Universities NHS Trust, 23Broomfield Hospital, 24 Royal Devon and Exeter NHS Foundation Trust, 25Bradford Teaching Hospitals NHS Foundation Trust, 26 The Psoriasis Association, 27St. John’s Institute of Dermatology, King’s College London, 28Institute of Infection, University of Glasgow, 29Dermatology Centre, University of Manchester, 30Institute of Translational and Clinical Medicine, University of Newcastle,31 National Amyloidosis Centre, University College London, 32 Department of Medical & Molecular Genetics, King’s College London.
British Journal of Dermatology. DOI: 10.111/bjd.20653

## Slide 2
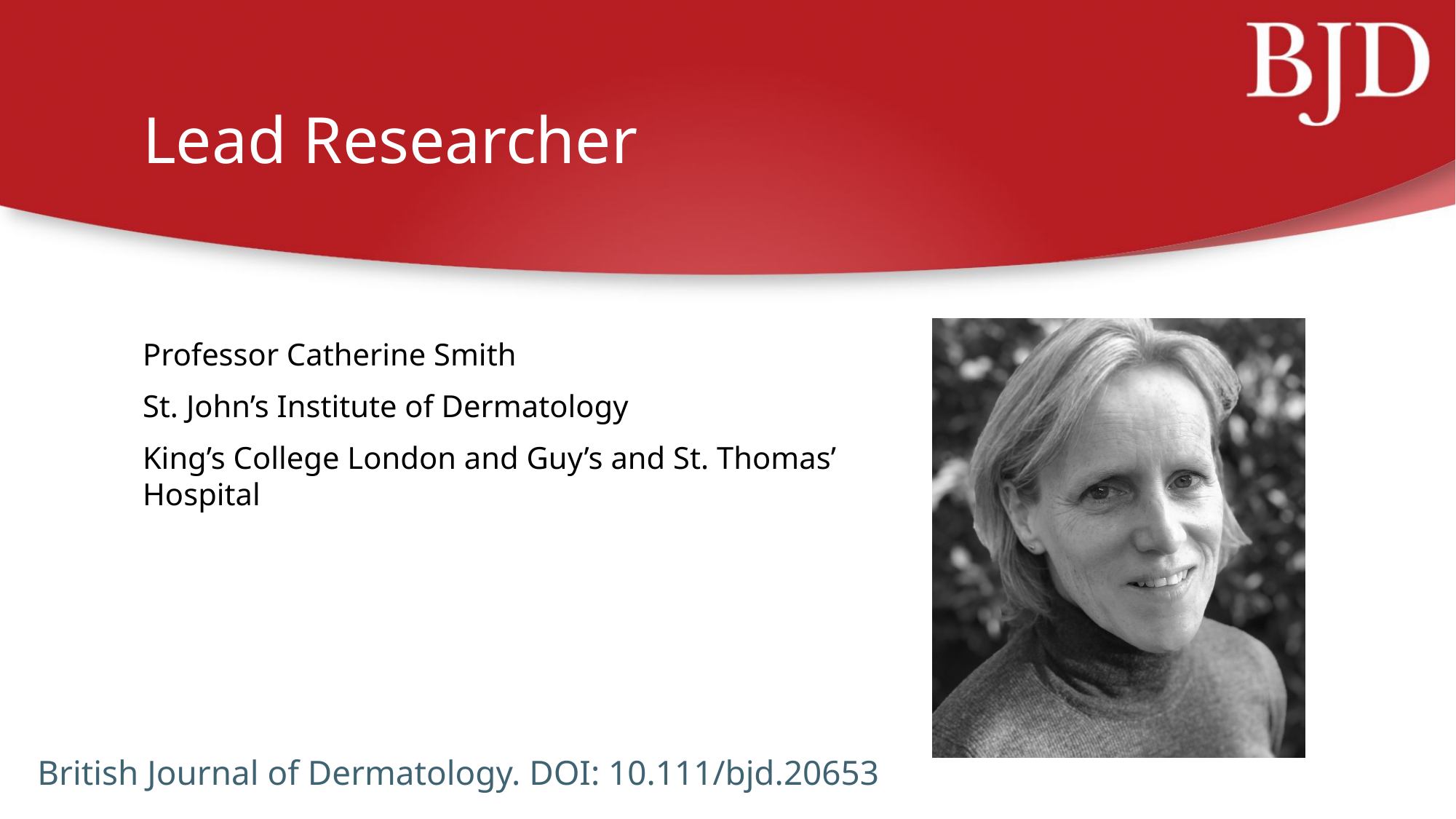

# Lead Researcher
Professor Catherine Smith
St. John’s Institute of Dermatology
King’s College London and Guy’s and St. Thomas’ Hospital
British Journal of Dermatology. DOI: 10.111/bjd.20653

## Slide 3
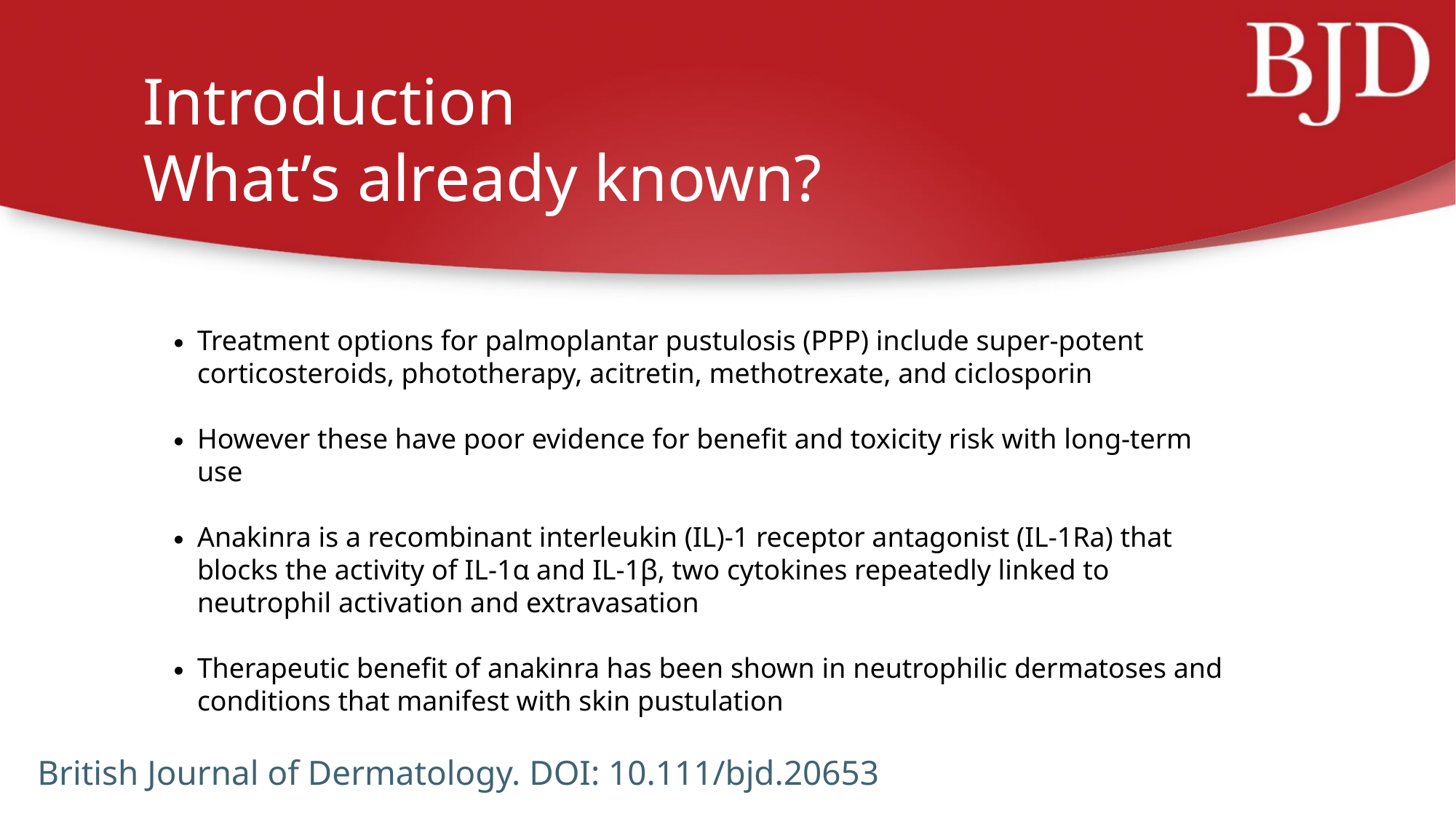

# Introduction What’s already known?
Treatment options for palmoplantar pustulosis (PPP) include super-potent corticosteroids, phototherapy, acitretin, methotrexate, and ciclosporin
However these have poor evidence for benefit and toxicity risk with long-term use
Anakinra is a recombinant interleukin (IL)-1 receptor antagonist (IL-1Ra) that blocks the activity of IL-1α and IL-1β, two cytokines repeatedly linked to neutrophil activation and extravasation
Therapeutic benefit of anakinra has been shown in neutrophilic dermatoses and conditions that manifest with skin pustulation
British Journal of Dermatology. DOI: 10.111/bjd.20653

## Slide 4
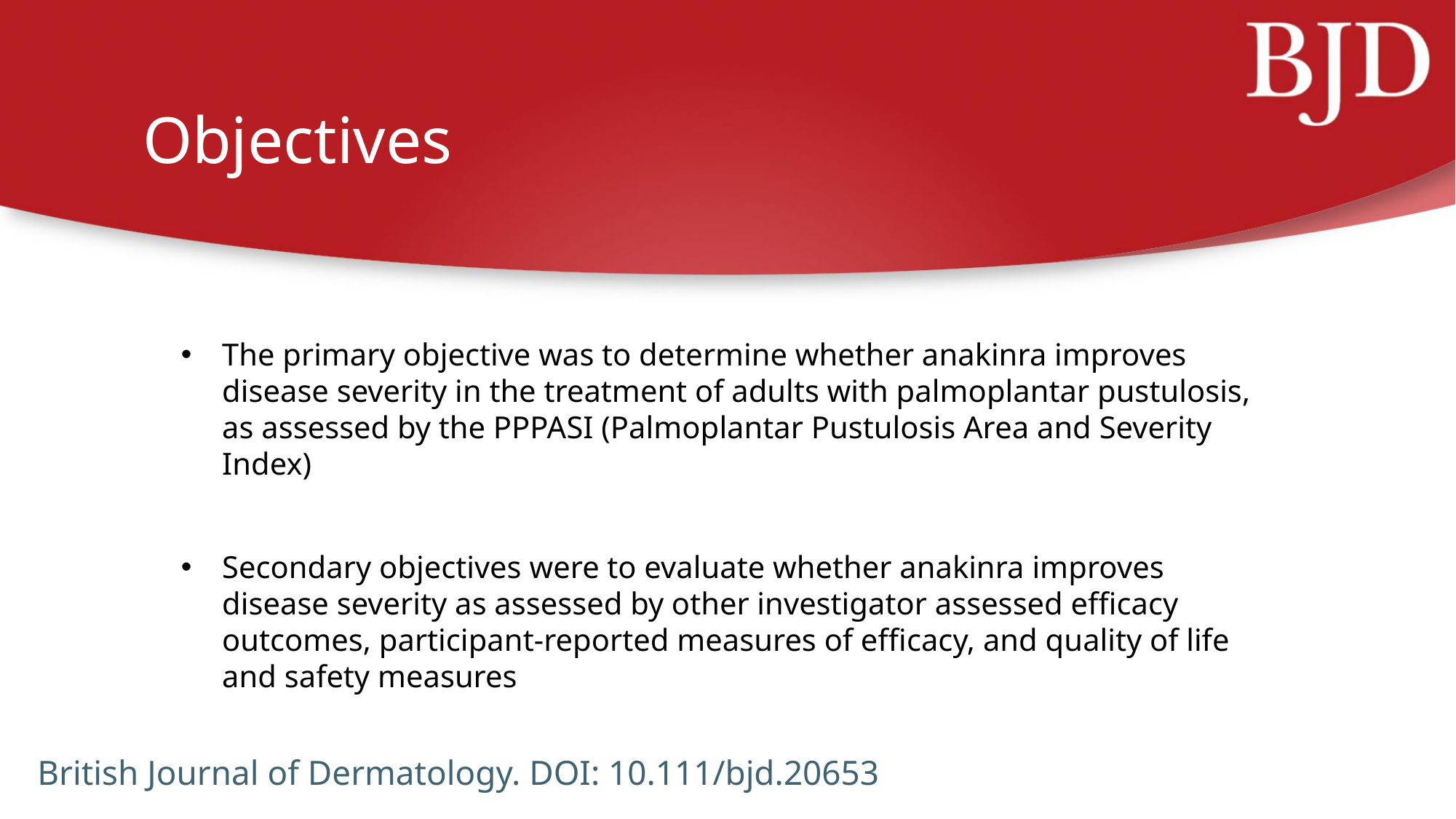

# Objectives
The primary objective was to determine whether anakinra improves disease severity in the treatment of adults with palmoplantar pustulosis, as assessed by the PPPASI (Palmoplantar Pustulosis Area and Severity Index)
Secondary objectives were to evaluate whether anakinra improves disease severity as assessed by other investigator assessed efficacy outcomes, participant-reported measures of efficacy, and quality of life and safety measures
British Journal of Dermatology. DOI: 10.111/bjd.20653

## Slide 5
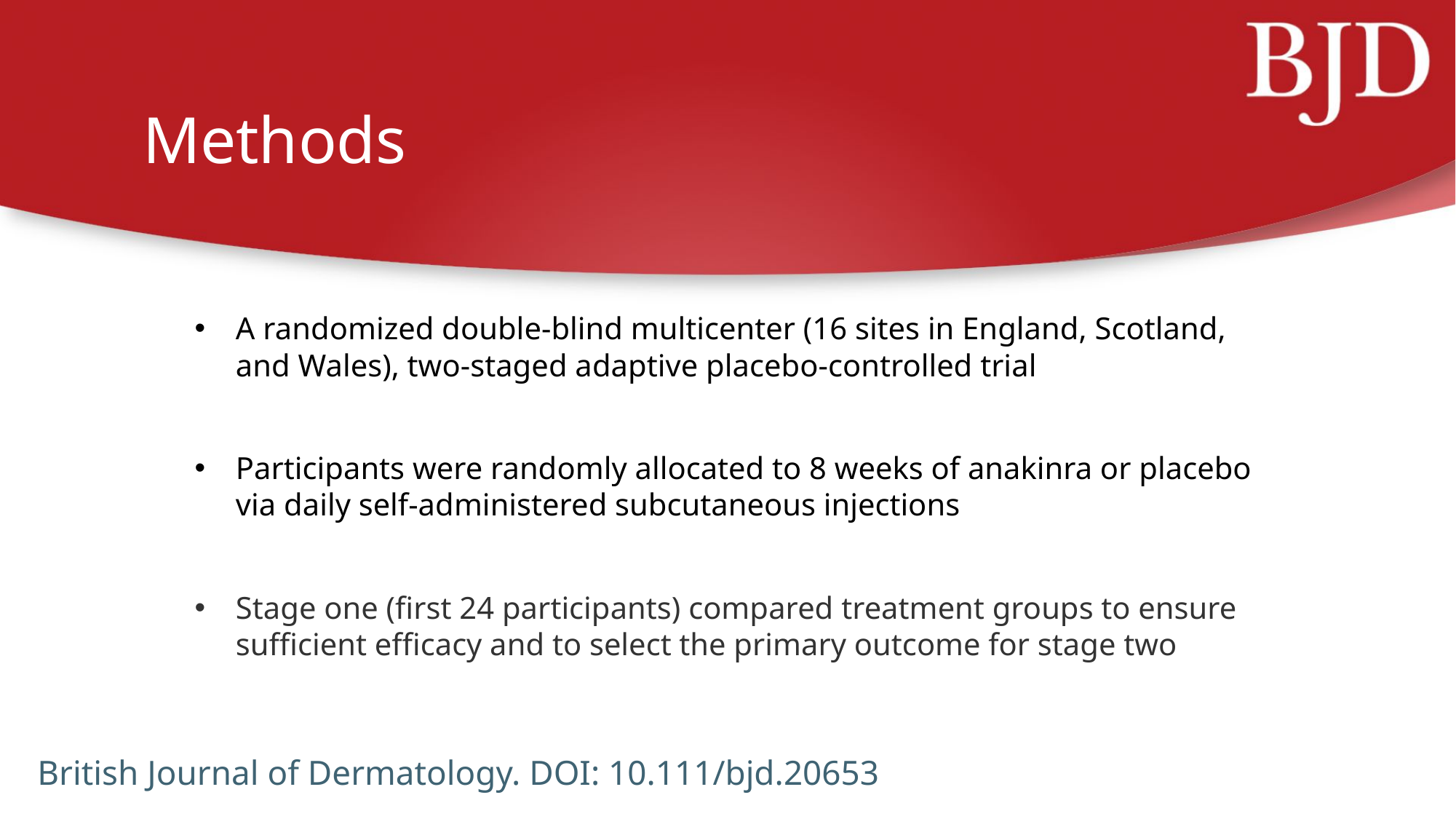

# Methods
A randomized double-blind multicenter (16 sites in England, Scotland, and Wales), two-staged adaptive placebo-controlled trial
Participants were randomly allocated to 8 weeks of anakinra or placebo via daily self-administered subcutaneous injections
Stage one (first 24 participants) compared treatment groups to ensure sufficient efficacy and to select the primary outcome for stage two
British Journal of Dermatology. DOI: 10.111/bjd.20653

## Slide 6
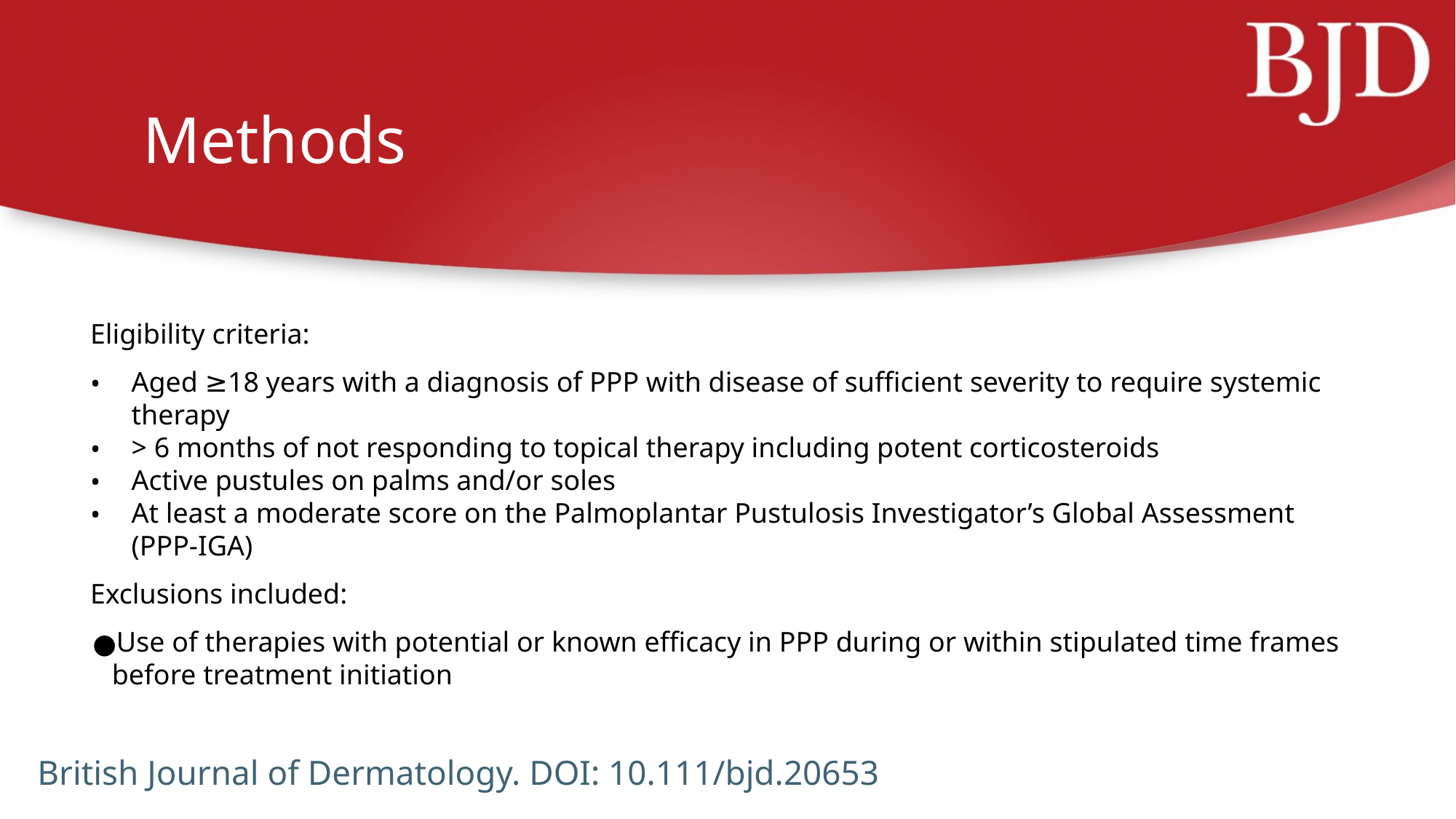

# Methods
Eligibility criteria:
Aged ≥18 years with a diagnosis of PPP with disease of sufficient severity to require systemic therapy
> 6 months of not responding to topical therapy including potent corticosteroids
Active pustules on palms and/or soles
At least a moderate score on the Palmoplantar Pustulosis Investigator’s Global Assessment (PPP-IGA)
Exclusions included:
Use of therapies with potential or known efficacy in PPP during or within stipulated time frames before treatment initiation
British Journal of Dermatology. DOI: 10.111/bjd.20653

## Slide 7
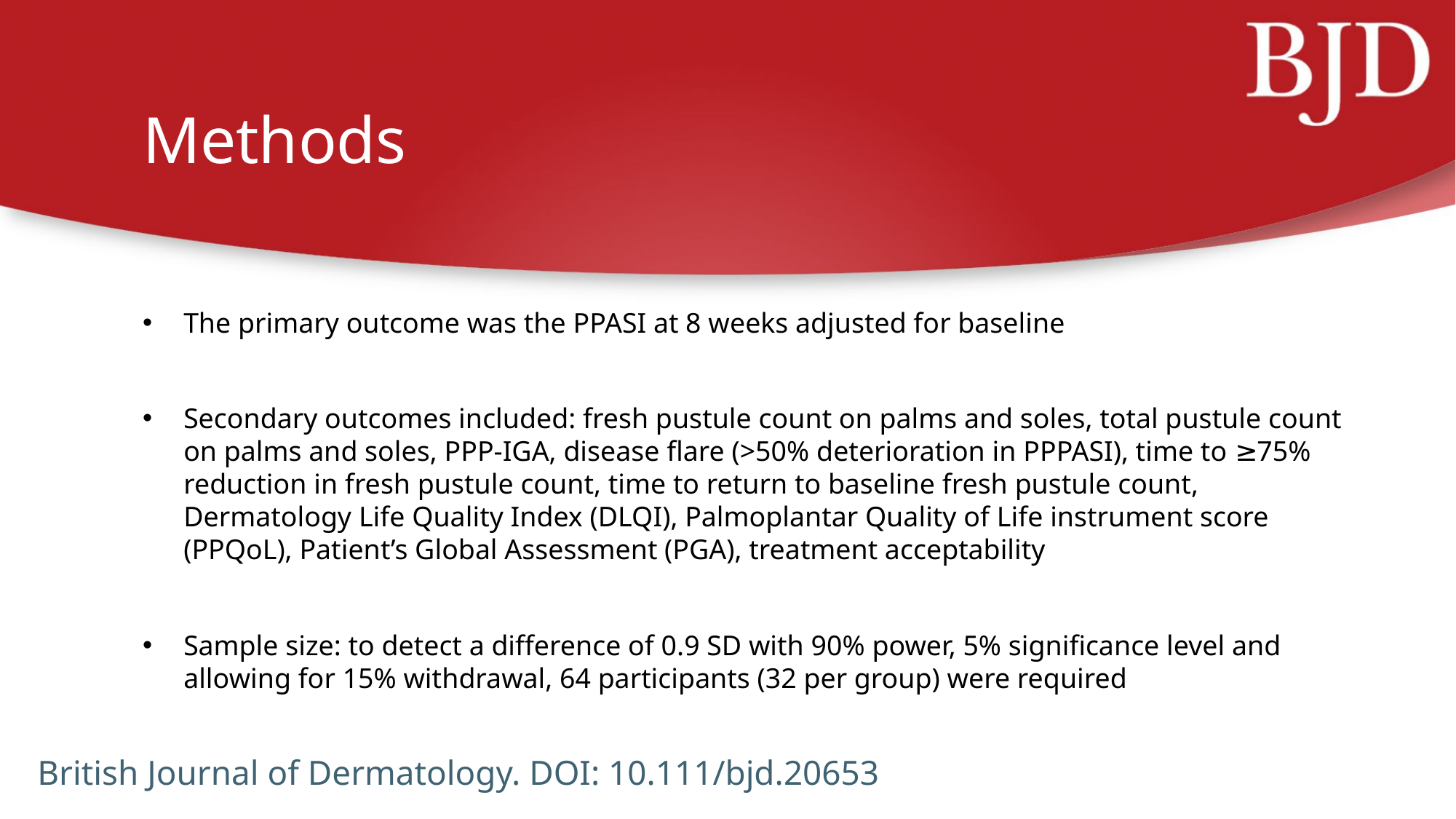

# Methods
The primary outcome was the PPASI at 8 weeks adjusted for baseline
Secondary outcomes included: fresh pustule count on palms and soles, total pustule count on palms and soles, PPP-IGA, disease flare (>50% deterioration in PPPASI), time to ≥75% reduction in fresh pustule count, time to return to baseline fresh pustule count, Dermatology Life Quality Index (DLQI), Palmoplantar Quality of Life instrument score (PPQoL), Patient’s Global Assessment (PGA), treatment acceptability
Sample size: to detect a difference of 0.9 SD with 90% power, 5% significance level and allowing for 15% withdrawal, 64 participants (32 per group) were required
British Journal of Dermatology. DOI: 10.111/bjd.20653

## Slide 8
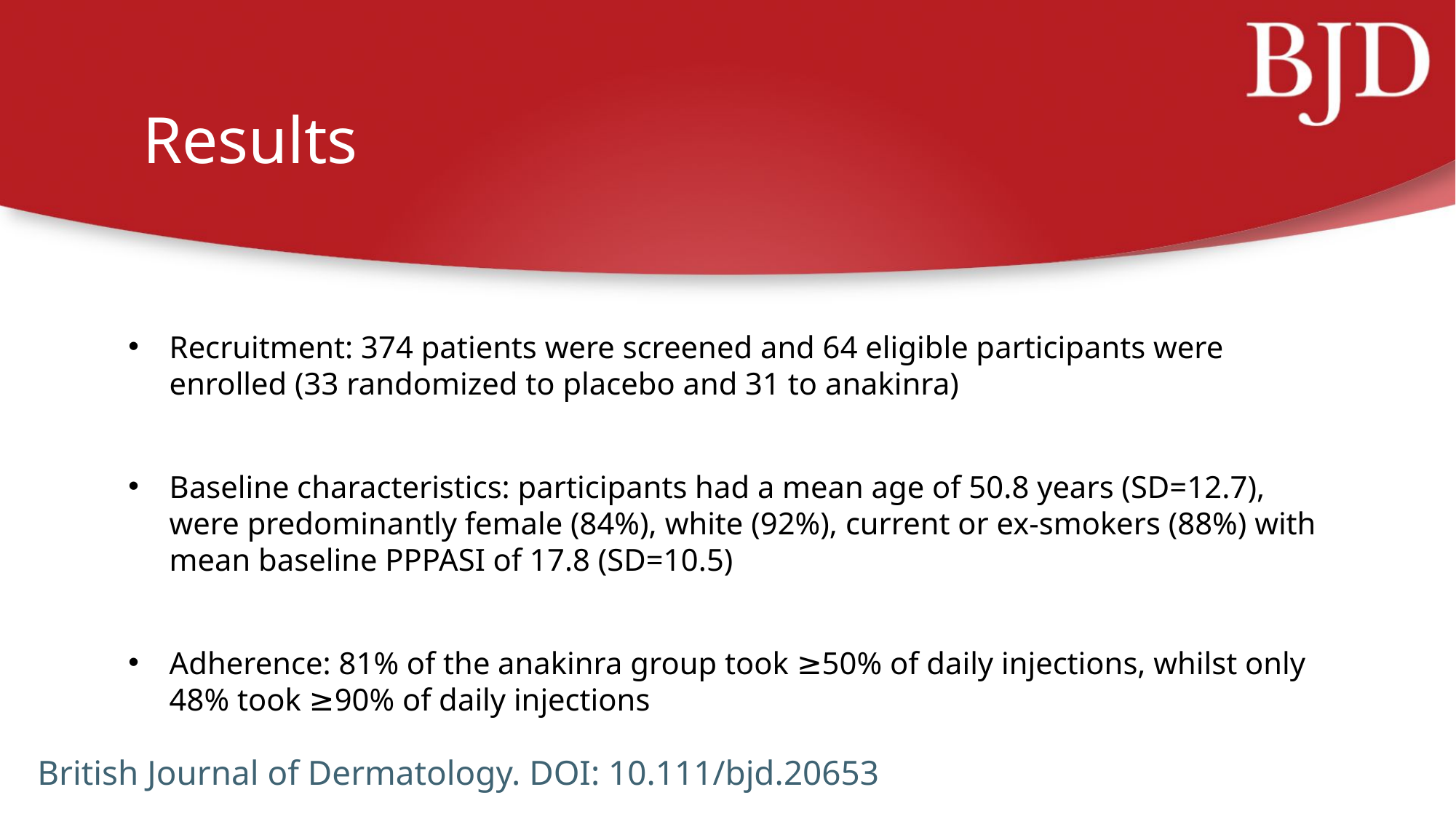

# Results
Recruitment: 374 patients were screened and 64 eligible participants were enrolled (33 randomized to placebo and 31 to anakinra)
Baseline characteristics: participants had a mean age of 50.8 years (SD=12.7), were predominantly female (84%), white (92%), current or ex-smokers (88%) with mean baseline PPPASI of 17.8 (SD=10.5)
Adherence: 81% of the anakinra group took ≥50% of daily injections, whilst only 48% took ≥90% of daily injections
British Journal of Dermatology. DOI: 10.111/bjd.20653

## Slide 9
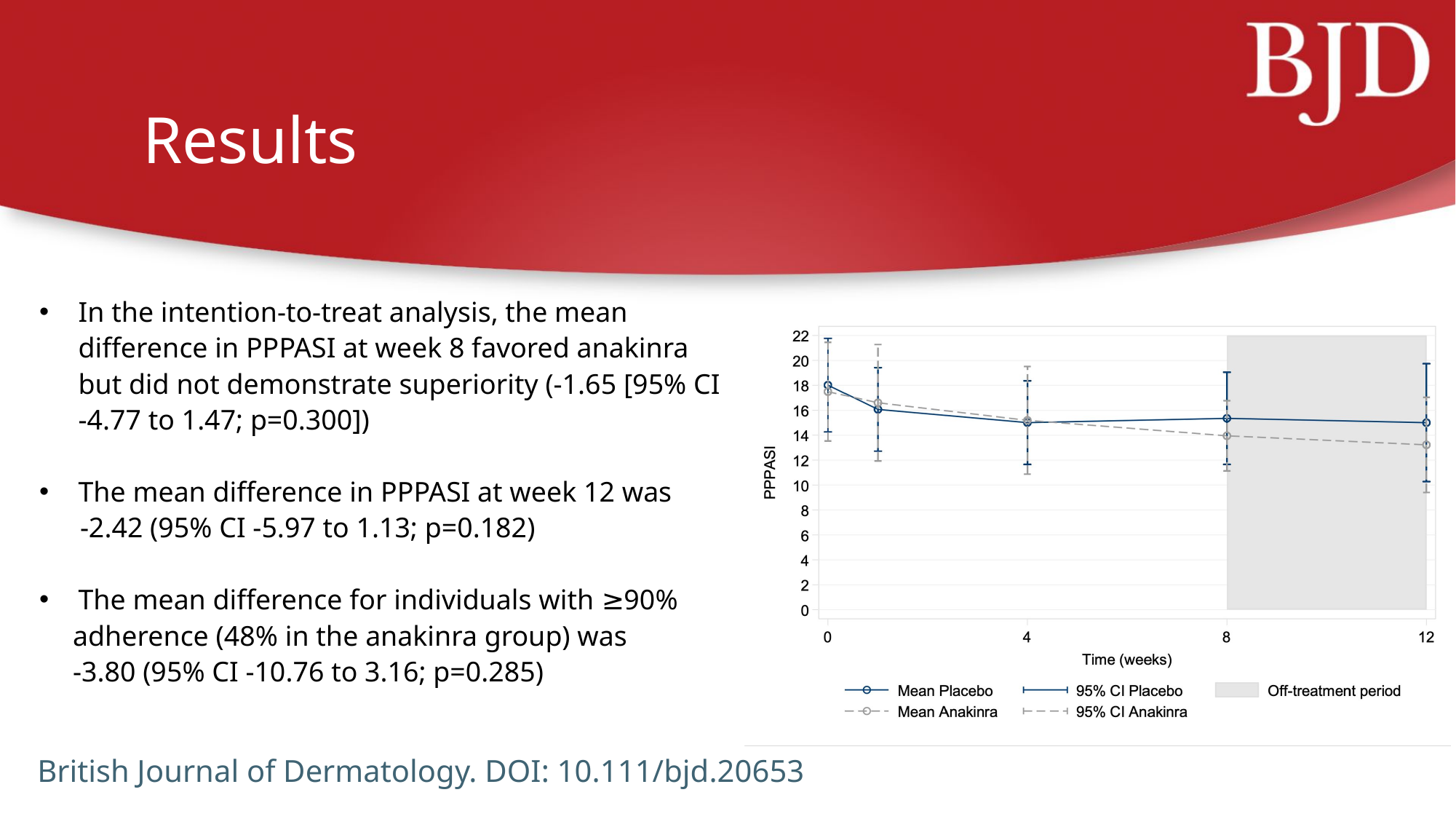

# Results
In the intention-to-treat analysis, the mean difference in PPPASI at week 8 favored anakinra but did not demonstrate superiority (-1.65 [95% CI -4.77 to 1.47; p=0.300])
The mean difference in PPPASI at week 12 was
 -2.42 (95% CI -5.97 to 1.13; p=0.182)
The mean difference for individuals with ≥90%
 adherence (48% in the anakinra group) was
 -3.80 (95% CI -10.76 to 3.16; p=0.285)
British Journal of Dermatology. DOI: 10.111/bjd.20653

## Slide 10
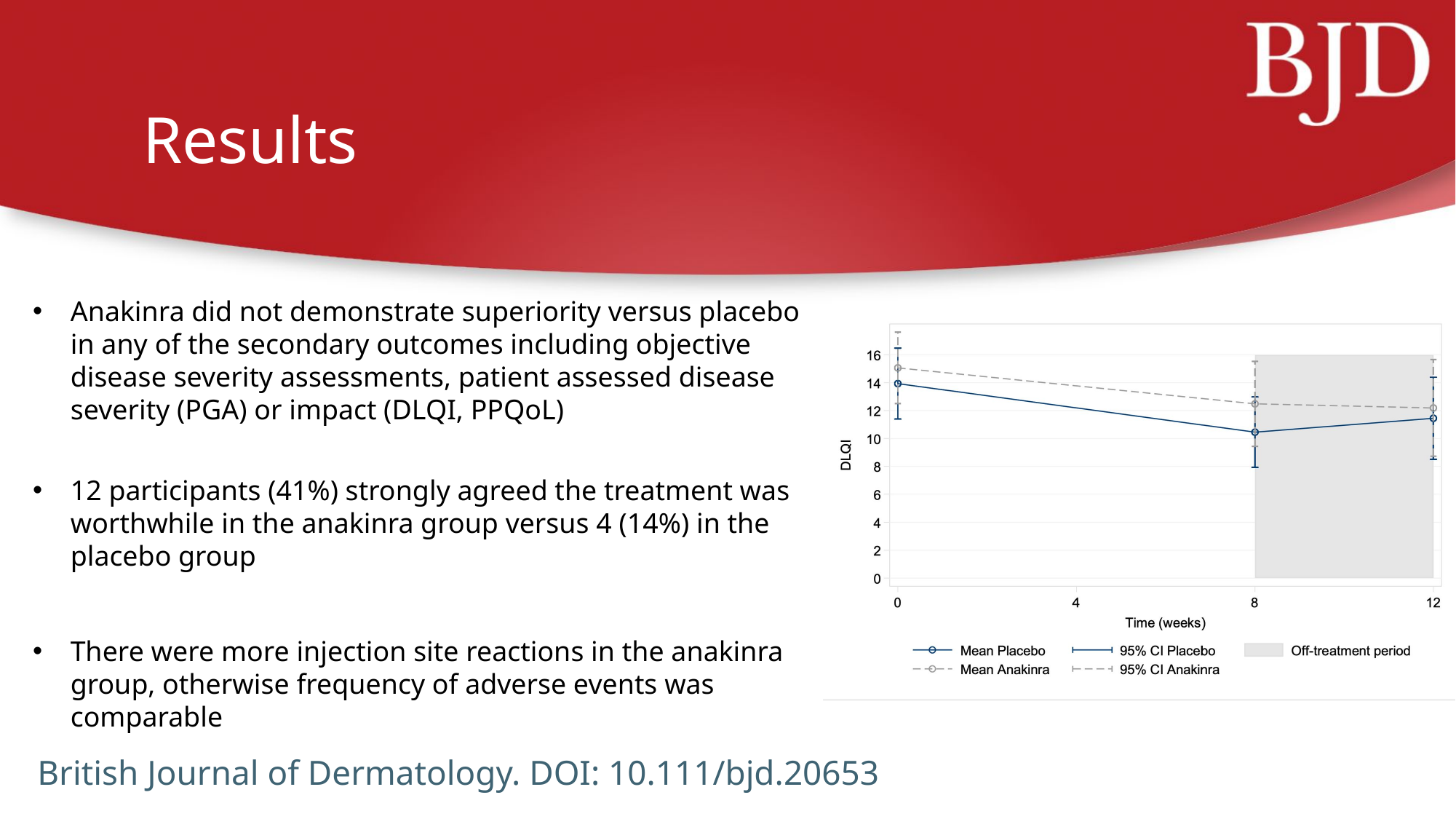

# Results
Anakinra did not demonstrate superiority versus placebo in any of the secondary outcomes including objective disease severity assessments, patient assessed disease severity (PGA) or impact (DLQI, PPQoL)
12 participants (41%) strongly agreed the treatment was worthwhile in the anakinra group versus 4 (14%) in the placebo group
There were more injection site reactions in the anakinra group, otherwise frequency of adverse events was comparable
British Journal of Dermatology. DOI: 10.111/bjd.20653

## Slide 11
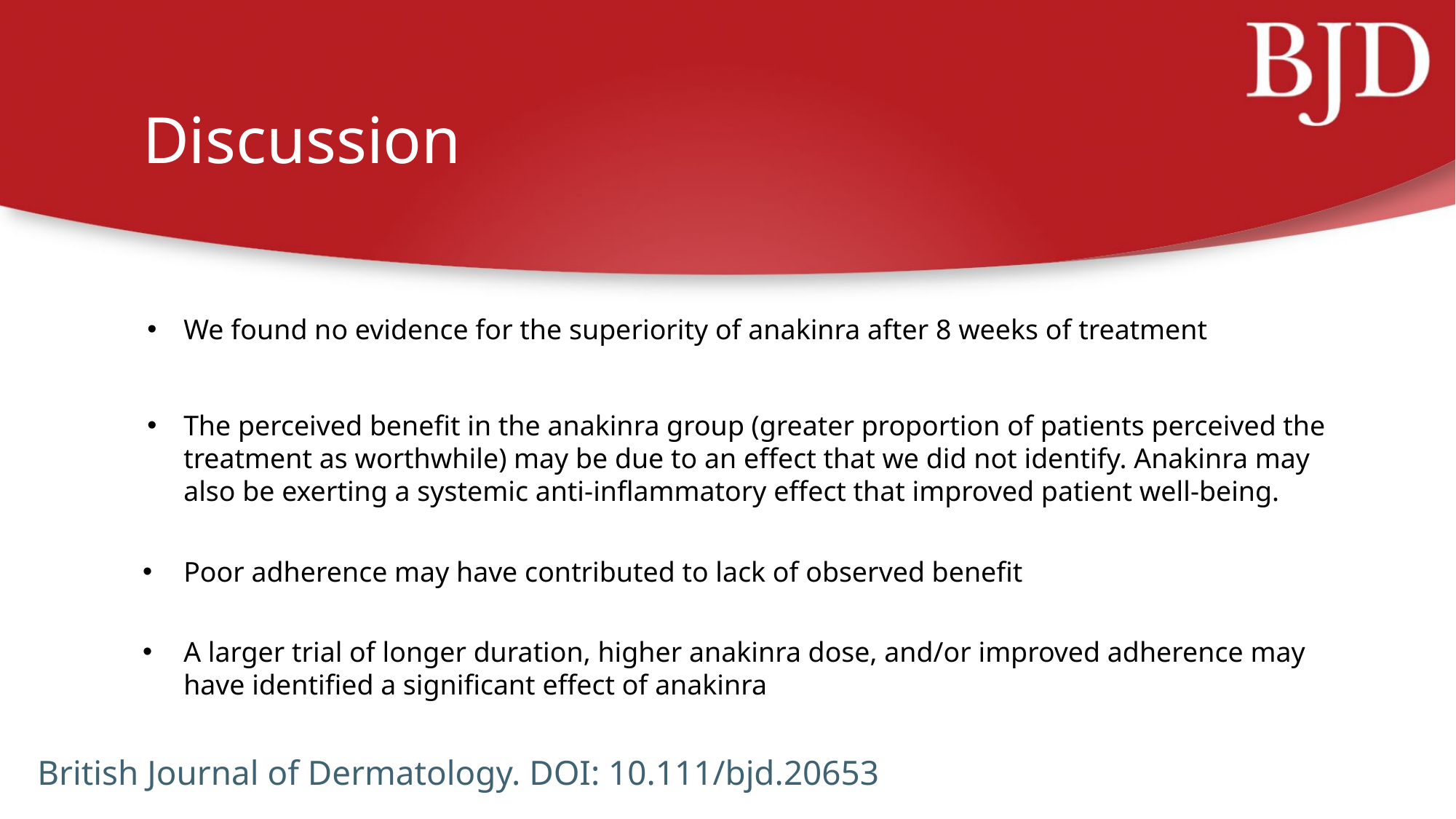

# Discussion
We found no evidence for the superiority of anakinra after 8 weeks of treatment
The perceived benefit in the anakinra group (greater proportion of patients perceived the treatment as worthwhile) may be due to an effect that we did not identify. Anakinra may also be exerting a systemic anti-inflammatory effect that improved patient well-being.
Poor adherence may have contributed to lack of observed benefit
A larger trial of longer duration, higher anakinra dose, and/or improved adherence may have identified a significant effect of anakinra
British Journal of Dermatology. DOI: 10.111/bjd.20653

## Slide 12
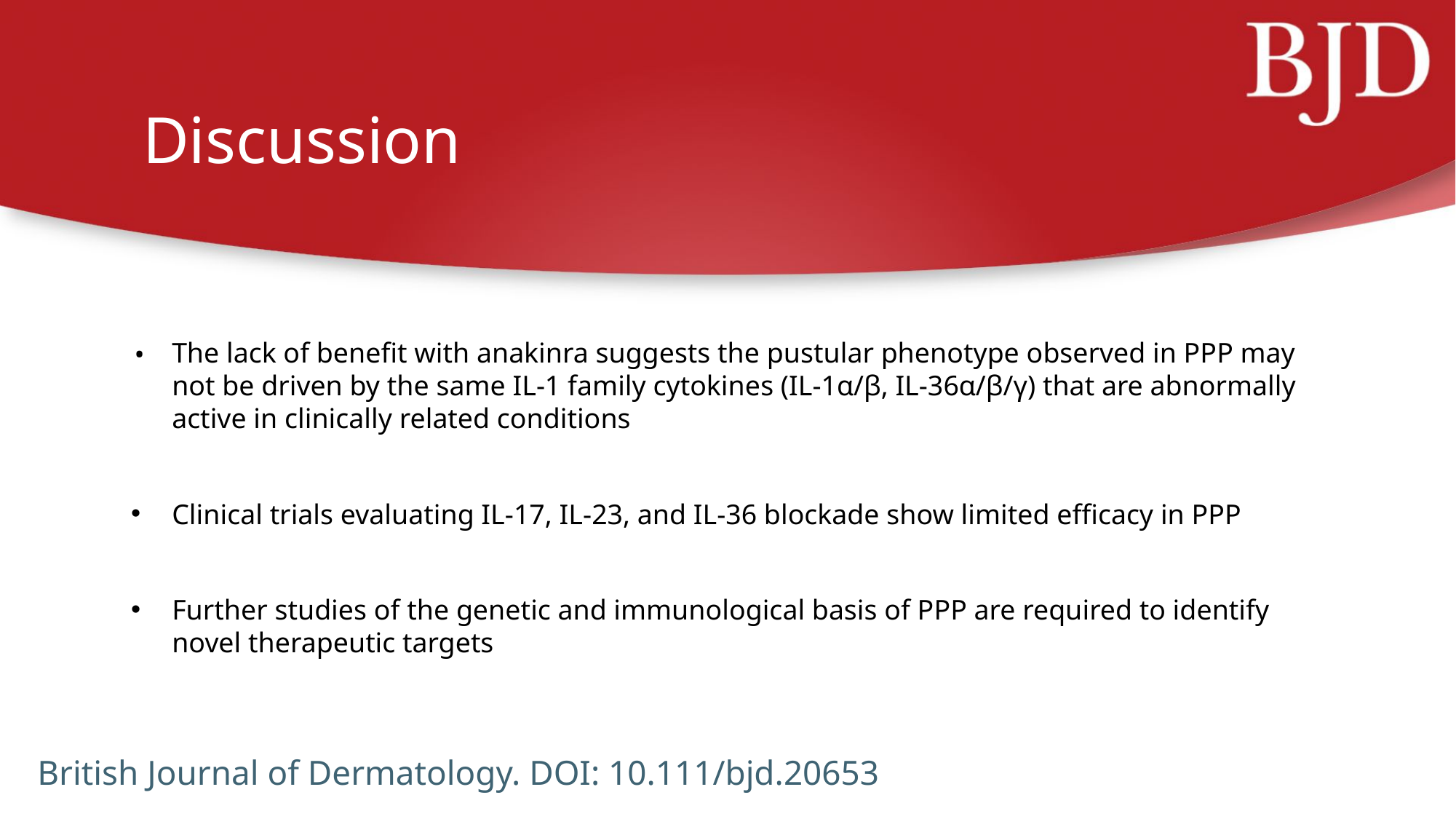

# Discussion
The lack of benefit with anakinra suggests the pustular phenotype observed in PPP may not be driven by the same IL-1 family cytokines (IL-1α/β, IL-36α/β/γ) that are abnormally active in clinically related conditions
Clinical trials evaluating IL-17, IL-23, and IL-36 blockade show limited efficacy in PPP
Further studies of the genetic and immunological basis of PPP are required to identify novel therapeutic targets
British Journal of Dermatology. DOI: 10.111/bjd.20653

## Slide 13
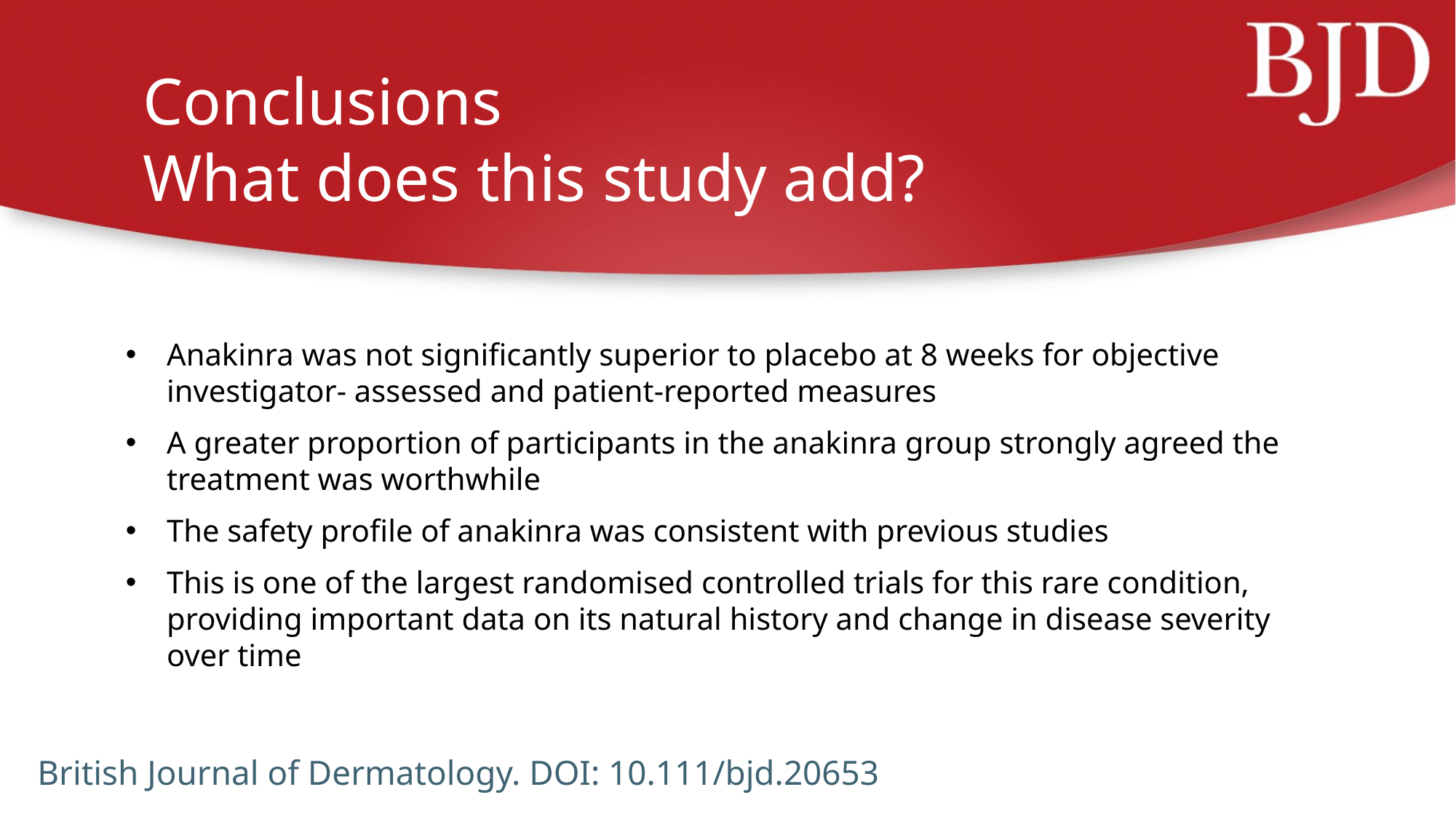

# Conclusions What does this study add?
Anakinra was not significantly superior to placebo at 8 weeks for objective investigator- assessed and patient-reported measures
A greater proportion of participants in the anakinra group strongly agreed the treatment was worthwhile
The safety profile of anakinra was consistent with previous studies
This is one of the largest randomised controlled trials for this rare condition, providing important data on its natural history and change in disease severity over time
British Journal of Dermatology. DOI: 10.111/bjd.20653

## Slide 14
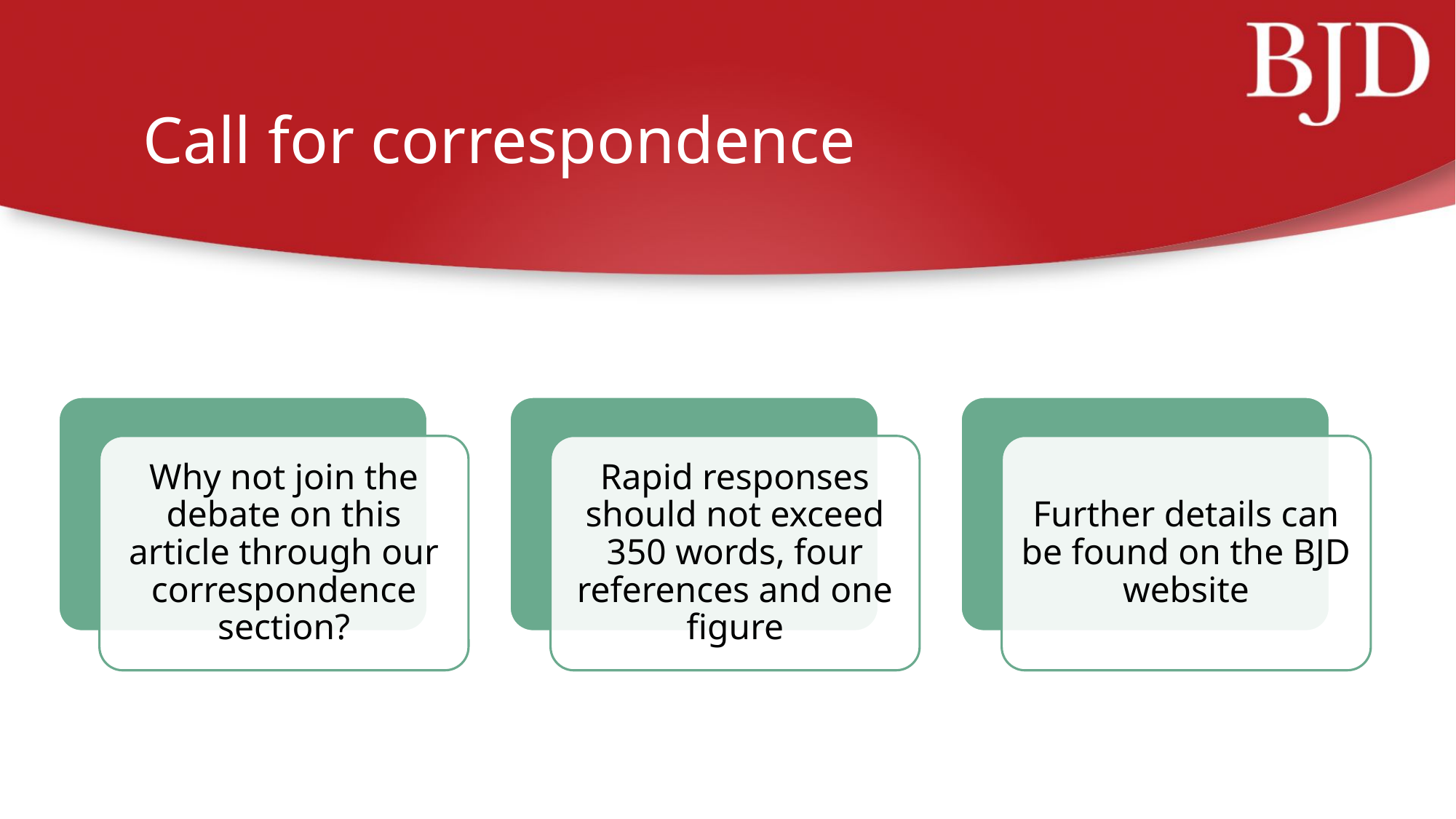

# Call for correspondence
Why not join the debate on this article through our correspondence section?
Rapid responses should not exceed 350 words, four references and one figure
Further details can be found on the BJD website
